# Extended-spectrum beta-lactamases in poultry in Africa: a systematic review

**DOI:** 10.3389/frabi.2023.1140750

**Published:** 2023-05-08

**Authors:** Akeemat O. Ayinla, Ana L. P. Mateus

**Affiliations:** ^1^ Royal Veterinary College, London, United Kingdom; ^2^ Department of Pathobiology and Population Sciences, Royal Veterinary College, London, United Kingdom

**Keywords:** Escherichia coli, Enterobacteria, ESBLs, antimicrobial resistance, AMR, poultry, systematic review, Africa

## Abstract

Extended-spectrum beta-lactamase (ESBL)-producing bacteria present a unique problem because of their ability to cause infections that are difficult to treat in animals and humans. The presence of ESBL-*Escherichia coli* (*E. coli*) in poultry raises a major public health concern due to the risk of zoonotic transfer *via* the food chain and direct contact with birds and the environment. This review aimed to determine the frequency of ESBL-producing *E. coli* and associated ESBL genes in poultry in Africa. Three databases (PubMed, ScienceDirect, and Web of Science) and predetermined websites were searched to identify scientific and grey literature. Studies (1582) were screened at title, abstract, and full-text levels. This review was registered with PROSPERO (CRD42021259872). Thirty-three studies were deemed eligible for this review. Phenotypic ESBL expression was confirmed in 22 studies (66.7%) with a wide range of colonization noted in sampled poultry (1 – 100%). The *bla* **_CTX-M_** gene was the most commonly isolated with the variants *bla* **_CTX-M-1_** and *bla* **_CTX-M-15_** being the most predominant in North and West Africa respectively. ESBL-producing *E. coli* isolates are frequently detected in poultry in farms and slaughterhouses across Africa thereby posing a potential health risk to humans. The paucity of data however does not allow for inferences to be made about the true extent of ESBLs in poultry in Africa.

## 1 Introduction

Extended-spectrum β-lactamase (ESBL)-producing bacteria pose a serious risk to both humans and animals (Mughini-Gras et al., 2019). The enzymes produced by these bacteria confer resistance to beta-lactam antibiotics such as first, second and third generation cephalosporins, penicillins, and monobactams, rendering them ineffective in treating infections caused by Gram-negative bacteria (Laube et al., 2013). ESBL-producing bacteria sometimes present resistance to antibiotics of other classes, further exacerbating treatment failure and increasing morbidity and mortality in affected individuals and animal populations alike (Rawat and Nair, 2010). The limited treatment options brought about by these resistant bacteria necessitates the use of ‘last resort’ antibiotics such as colistin and carbapenems, thereby promoting resistance to these drugs (Cantón et al., 2012).

ESBLs are most commonly produced by Enterobacteriaceae, especially *Escherichia coli* (*E. coli*) and *Klebsiella* spp. which have often been isolated in poultry (Blanc et al., 2006; Li et al., 2015). Based on their amino acid sequence, ESBLs can be classified into nine evolutionary and structural families: TEM, SHV, CTX-M, VEB, GES, BES, PER, TLA, and OXA (Paterson and Bonomo, 2005), out of which TEM, CTX-M, SHV, and OXA are the major groups often utilized for the molecular detection of ESBL genes (Tängdén et al., 2010; Ur Rahman et al., 2018). CTX-M represents the most widespread type of ESBLs isolated from humans and poultry, but the predominant variants of this gene differ in both populations (Platell et al., 2011; Gundran et al., 2019). CTX-M-14 and CTX-M-15 are the major variants observed in humans regardless of geography. In poultry, CTX-M-1 is most commonly isolated in Europe while CTX-M-14 is most common in Asia (Ewers et al., 2012). However, there are dissenting opinions on predominant variants of CTX-M in poultry in Africa (Alonso et al., 2017; Meguenni et al., 2019).

The use of antibiotics, including beta-lactams, in animal husbandry has been linked to an increased occurrence of resistant bacteria in food-producing animals. The rise in consumer demand for poultry products in low- and middle-income countries (LMICs), including African countries, and a transition to large-scale intensive production systems led to an increase in antibiotic use (ABU) in food-producing animals (Klein et al., 2018). Worryingly, the inadequate biosecurity and poor hygiene and sanitation in poultry production systems in LMICs has resulted in a high reliance on ABU for disease prevention and control (Hedman et al., 2020). Higher incidence of drug-resistant bacteria including ESBL-producing bacteria has been reported in poultry production systems with high levels of ABU (ben Sallem et al., 2012; Donkor et al., 2012).

Since the earliest detection of ESBLs in healthy poultry between 2000 and 2001 in Spain by Briñas et al. (2003), ESBL-producing bacteria have been isolated in poultry in many countries including African countries (Overdevest et al., 2011; Blaak et al., 2015; Maamar et al., 2016; Brower et al., 2017; Aworh et al., 2020). The detection of ESBL-producing *E. coli* in healthy poultry is a problem due to the potential risk of zoonotic spread to human populations *via* the food chain and the risk of causing severe illness and a burden on healthcare services due to prolonged hospitalization periods in affected individuals (Olsen et al., 2014; Ramos et al., 2020). The detection of the same ESBL genes, such as *bla*_CTX-M-15_, and closely related ESBL-producing *E. coli* isolate clusters in poultry and humans further suggests the spread of these bacteria between both poultry and human populations (Dierikx et al., 2013; Falgenhauer et al., 2019).

The likelihood of interhost transfer of resistance between poultry and humans appears to be relatively higher in some areas in Africa where people often live in close contact with poultry (Alonso et al., 2017). Moreover, ABU in many of these settings remains mostly unregulated (Maron et al., 2013). Currently, major data gaps exist in Africa with respect to the true burden of AMR; strengthening the evidence base is germane for the development of effective interventions to tackle AMR in this continent (Elton et al., 2020). The purpose of this study is to determine the frequency of ESBL-producing *E. coli* and the most prevalent ESBL genes in poultry in farms and slaughterhouses across the African continent. Additionally, the study identified the predominant methods of ESBL testing and the gaps in the knowledge of ESBLs in poultry in African countries.

## 2 Materials and methods

### 2.1 Search procedure

Three databases: Science Direct, PubMed, and Web of Science were searched (from January 1, 2000, until May 24 2021) for studies relevant to the review, using a combination of words from four groups of search terms relating to “poultry”, “ESBL”, “antibiotic resistance” and “Africa” (**Supplementary Table S1**). Predetermined websites of national and international organizations (World Health Organisation (WHO), Food and Agricultural Organization of the United Nations (FAO), World Organisation for Animal Health (WOAH, founded as OIE), International Livestock Research Institute (ILRI), Africa Centre for Disease Control and Prevention (CDC), World Bank, World Food Bank, and African Union) were also searched to identify relevant grey literature. The study protocol was submitted and registered with the PROSPERO International Prospective Register of Systematic Reviews (study ID: CRD42021259872) before the start of the literature search in April 2021.

### 2.2 Inclusion criteria

This review included: (i) observational studies that assessed ESBL-producing *E. coli* and in which ESBL genes were detected in fecal samples, cloacal swabs, and cecal content from domestic poultry (chickens and turkeys) at farm and slaughterhouse levels and, (ii) studies that were carried out in African countries and published in English language between 2000 and 2021. In studies where different resistance patterns and/or genes were investigated, only data related to ESBLs was extracted. In studies that sampled domestic poultry of interest as well as other species, only data related to poultry was extracted.

### 2.3 Exclusion criteria

This review excluded: (i) studies that considered bacteria species other than *E. coli*, only pathogenic *E. coli* or non-ESBL producing *E. coli*, (ii) studies that focused solely on humans, environment and/or animal species other than poultry, (iii) studies that assessed ducks, geese, and wild birds, (iv) studies where only poultry-derived products, poultry meat, carcasses, or internal organs were sampled, (v) reviews, abstracts from conference proceedings, chapters from books or textbooks, and studies for which the full text could not be obtained.

### 2.4 Study screening

All studies identified using the search strategy were imported into a reference management software (Mendeley version 1.19.4) and the duplicates were removed. Screening of the studies against the eligibility criteria was carried out in three steps at title, abstract, and full text levels.

### 2.5 Citation and reference tracking

Citation and reference tracking of eligible publications was conducted to identify previously unidentified studies relevant to the systematic review. This was done simultaneously with data extraction.

### 2.6 Study quality and risk of bias assessment

The quality and risk of bias assessment was carried out using an adapted version of the quality assessment tool described by Sargeant et al. (2005). A checklist was used to appraise each study based on study objectives and population, outcome assessment, data analysis, results, and conclusions (**Supplementary Table S2**). Every study was appraised by assigning a score for each item on the checklist and adding up the total scores. The cumulative scores for each study were then interpreted using a rating scale of 0-16. Studies with scores between 0-5 were grouped as low quality, those with scores between 6-11 were termed intermediate quality while high-quality studies were defined as those with a score of 12-16.

### 2.7 Data extraction

An Excel data extraction template was created (Microsoft Excel, version 16.0). The column headings were defined in line with the research questions and eligibility criteria. The data extracted included general details about the publication (author, publication date and period of study, country), study objectives or research questions, study characteristics (study design, sampling strategy, and sampling size), the population (species, type of production system, age, health status), exposure (number of *E. coli* isolates) and outcome of interest (methods of ESBL detection, ESBL frequency, and percentage, ESBL genes). In this study, ESBL percentage refers to the number of ESBL-producing *E. coli* out of the total number of *E. coli* isolates tested.

The different types of production systems (i.e., intensive, semi-intensive, extensive, small scale, household/backyard) were collated by the authors when these were reported in the studies. In studies where only the farm population has been stated, farms with less than 200 birds were classified as small scale, while those with 200 – 1,000 birds were classified as medium scale and farms with over 1,000 birds were classified as large-scale production systems following the FAO’s classification of poultry operations (FAO, 2014).

### 2.8 Data analysis and synthesis

Due to the heterogeneity of the data, a meta-analysis could not be carried out in this review. Instead, a narrative analysis of the study characteristics was conducted with the use of tables and figures. Studies were grouped into three geographical regions: Northern, Western and Eastern Africa according to the United Nations geoscheme (UN, 2011).

## 3 Results

**Figure 1:**
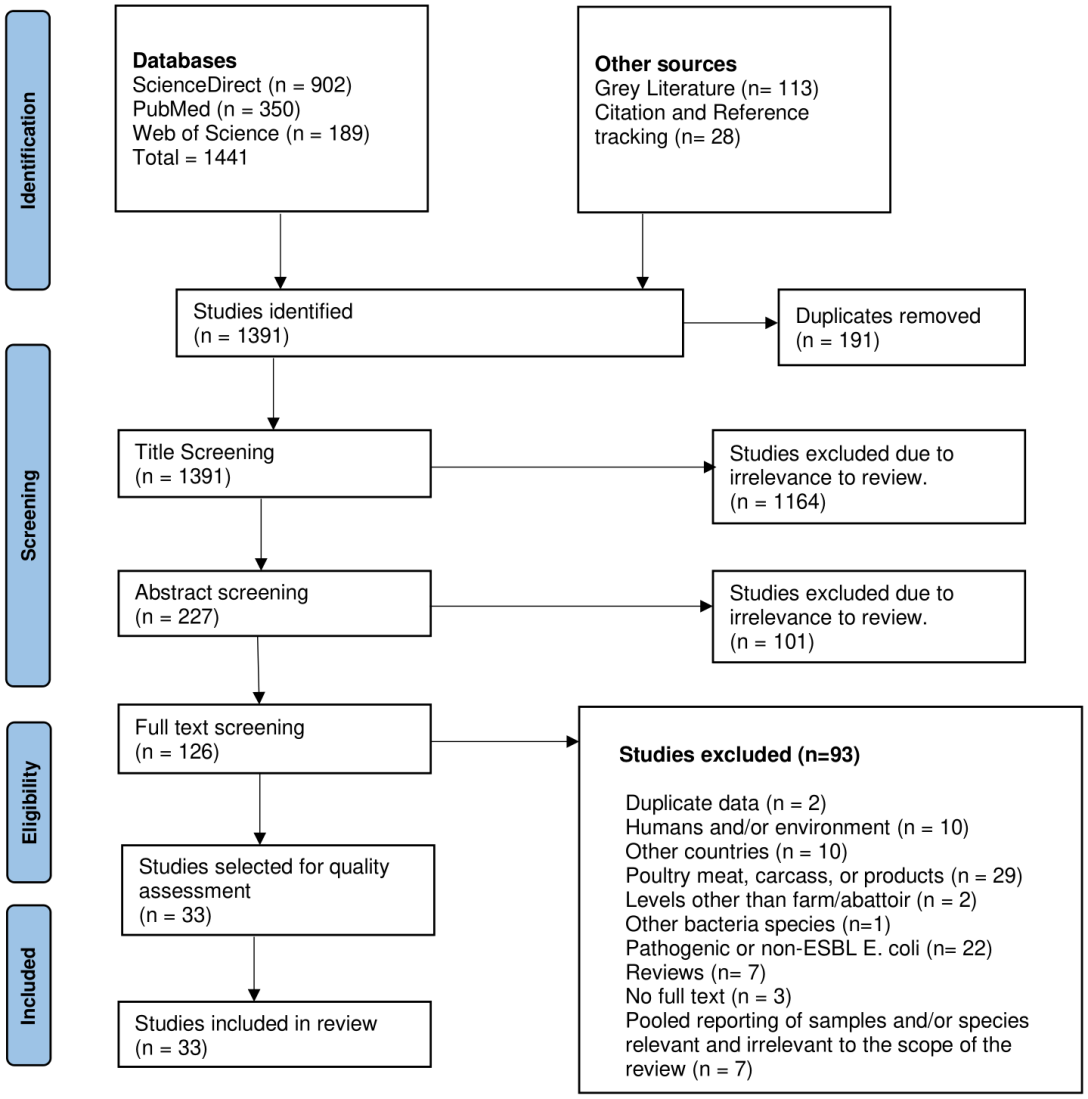
PRISMA flow chart of the study screening process conducted for this review (adapted from Page et al. (2021).

Overall, 1,441 studies were identified through scientific databases; 113 were identified from grey literature websites, and an additional 28 were identified through citation and reference tracking. After a three-tier screening at title, abstract and full text levels, a total of 33 studies were deemed eligible for inclusion in this review (**Figure 1**).

### 3.1 Quality of studies

Since most of the studies (27 studies, 81.8%) included in this review employed non-probabilistic sampling, failed to justify sample size, and did not address confounding, they were considered to be of moderate quality with moderate risk of bias. Only six studies were categorized as high quality with low risk of bias because they provided adequate description of the phenotypic and genotypic methods employed in the study, utilized appropriate statistical analysis, and the conclusions were logical and consistent with the findings of the study (**Table 1**).

**Table 1:** Study Quality and Risk of Bias Assessment of the 33 eligible studies.

Study details	Study Objectives (0 – 2)	Sample size justification (0 – 2)	Phenotypic Detection (0 – 2)	Genotypic Detection (0 – 2)	Statistical Analysis (0 – 2)	Confounding/	Results (0 – 2)	Conclusions (0 – 2)	Total (0 – 16)	Quality/
						Limitations (0 – 2)				Risk of Bias
Agabou et al., 2016	2	0	2	2	2	0	2	2	12	++
Aworh et al., 2020	2	0	1	2	2	0	1	2	10	+
Ayandiran et al., 2018	2	0	2	2	0	1	2	2	11	+
Ayeni et al., 2015	2	0	2	2	0	0	2	2	10	+
Badi et al., 2018	2	0	2	2	0	0	2	2	10	+
Belmahdi et al., 2016	2	0	2	2	0	0	2	2	10	+
ben Sallem et al., 2012	2	0	2	2	0	0	1	2	9	+
Büdel et al., 2020	2	0	1	2	0	0	1	2	8	+
Chabou et al., 2018	2	0	2	2	0	0	1	2	9	+
Chah et al., 2018	2	0	2	2	0	0	2	2	10	+
Falgenhauer et al., 2019	2	0	1	2	0	1	1	2	9	+
Fortini et al., 2011	2	0	2	2	0	0	2	0	8	+
Grami et al., 2013	2	0	2	2	0	0	1	2	9	+
Hassen et al., 2020	2	0	2	2	0	0	2	2	10	+
Jouini et al., 2007	2	0	2	2	0	0	1	2	9	+
Katakweba et al., 2018	2	0	2	2	0	0	2	2	10	+
Kilani et al., 2015	2	0	2	2	0	0	1	2	9	+
Kilani et al., 2020	0	0	2	2	0	0	1	2	7	+
Kimera et al., 2021	2	1	2	2	2	0	1	1	11	+
Kwoji et al., 2019	2	0	2	2	2	0	2	2	12	++
Langata et al., 2019	2	1	2	2	0	0	1	0	8	+
Maamar et al., 2016	2	0	2	2	2	0	2	2	12	++
Messaili et al., 2019	2	0	2	2	2	0	2	2	12	++
Mgaya et al., 2021	2	0	2	2	2	0	2	2	12	++
Mnif et al., 2012	2	0	2	2	0	0	2	2	10	+
Moawad et al., 2018	2	0	2	2	0	0	2	2	10	+
Ojo et al., 2016	2	0	2	2	0	0	2	2	10	+
Okpara et al., 2018	2	0	1	2	2	0	1	2	10	+
Okubo et al., 2019	2	0	2	2	0	1	2	2	11	+
Ramadan et al., 2018	2	0	2	2	2	0	1	2	11	+
Saidani et al., 2019	2	0	2	2	0	0	2	2	10	+
Sghaier et al., 2019	2	0	2	2	0	0	1	2	9	+
Vounba et al., 2019	2	2	2	2	2	1	1	2	14	++

+ Moderate quality/moderate risk of bias.

++ High quality/low risk of bias.

### 3.2 Study characteristics

#### 3.2.1 Location and timeline

**Figure 2:**
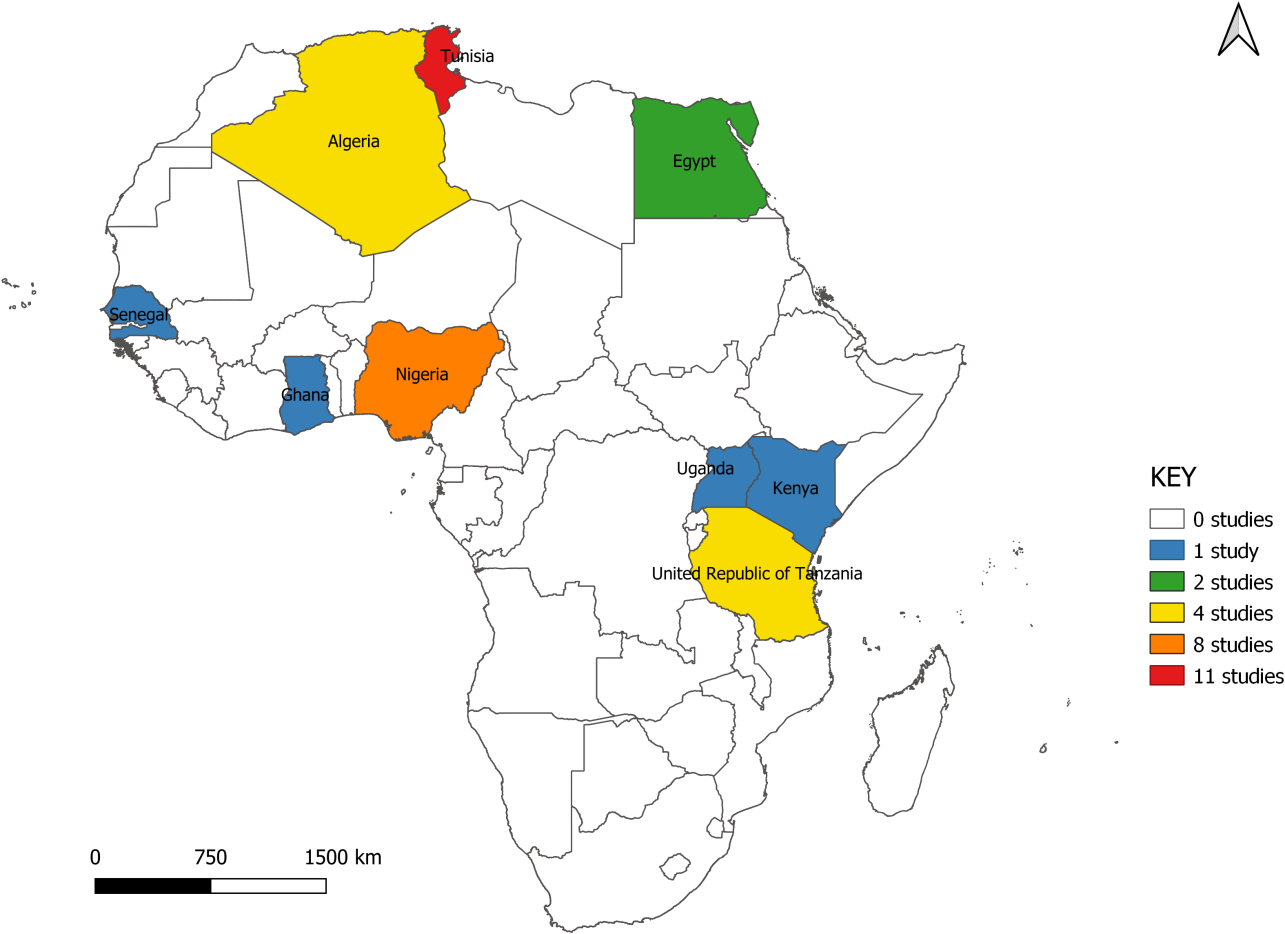
Map of study locations.

**Figure 3:**
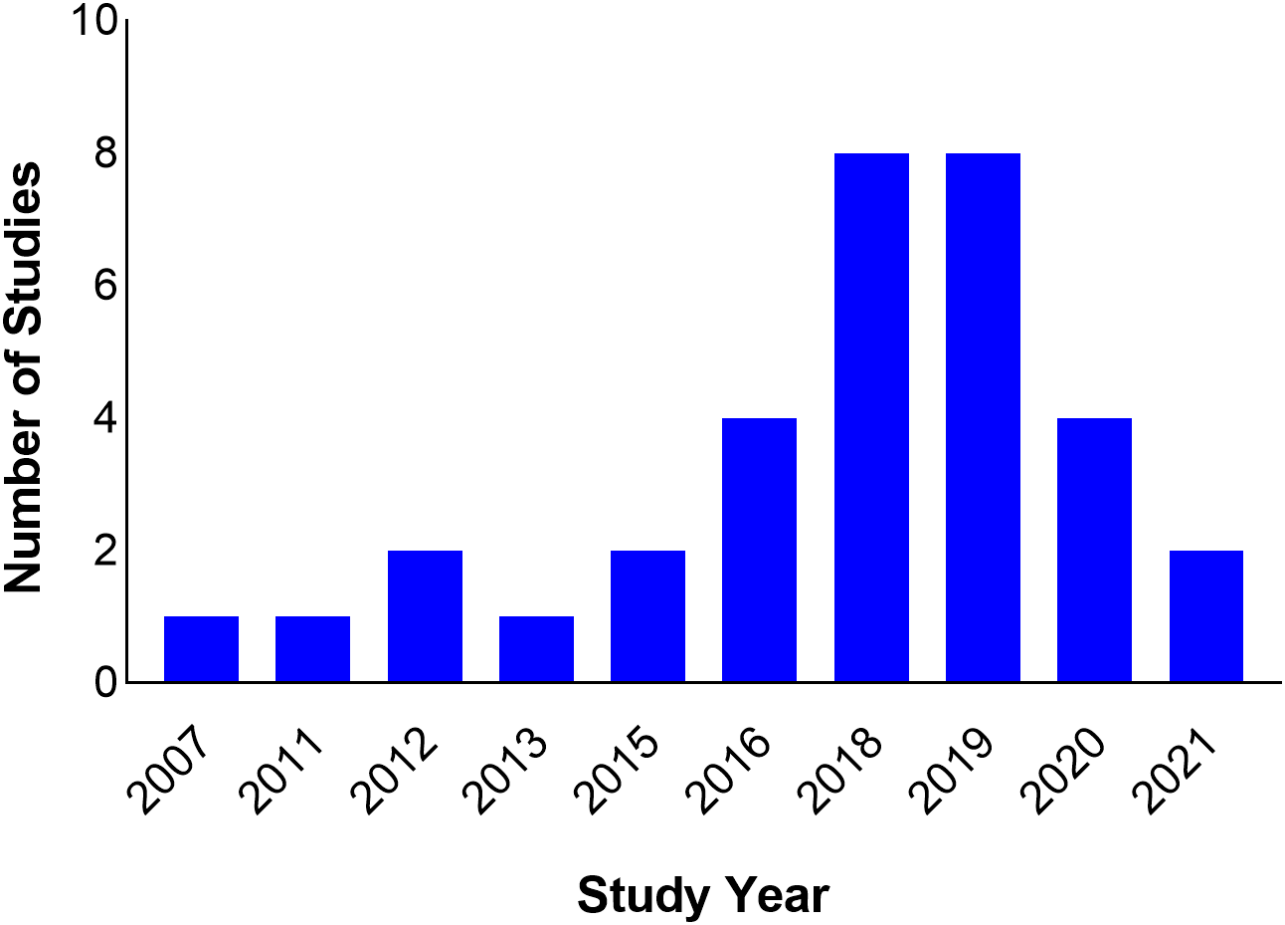
Timeline of study publications.

The studies span nine countries from North, West, and East Africa. Seventeen (51.5%) of the studies were carried out in North Africa (Algeria = 4, Egypt = 2, Tunisia = 11), six in East Africa (Kenya = 1, Tanzania = 4, Uganda = 1) and 10 in West Africa (Ghana = 1, Nigeria = 8, Senegal = 1) (**Figure 2**). All studies were scientific articles published between 2007 and 2021 with most of the studies (26 studies, 78.8%) published between 2016 and 2021. There was a decline in the number of publications from 2020 onwards (**Figure 3**).

#### 3.2.2 Study design and sampling strategy

All studies were cross-sectional studies. In one study (Falgenhauer et al., 2019), a repeat cross-sectional study design was employed, and results were reported as an overall proportion. With respect to sampling strategy, only two studies (Okpara et al., 2018; Vounba et al., 2019) employed probabilistic sampling, five used non-probabilistic sampling [purposive = 1 (Mgaya et al., 2021), convenience sampling = 4 (Chabou et al., 2018; Aworh et al., 2020; Hassen et al., 2020; Kimera et al., 2021)]; nevertheless, the majority of the studies (26 studies, 78.8%) did not provide any details of the sampling strategy used (**Table 2**).

**Table 2:** Characteristics of eligible studies.

Publication Details (Study period)	Location	Study design/Sampling strategy	Setting	Production system	Population	Age	Health Status	Sample size	Samples collected	Antibiotic Use/Purpose	Resistance Detection method
Agabou et al., 2016 (2011-2012)	Algeria	Cross-sectional/NR	Farm	NR	Chicken	≥ 35 days (5 weeks)	NR	280	Fecal sample	NR	Both*
Aworh et al., 2020 (2018-2019)	Nigeria	Cross-sectional/Convenience sampling	Farm	NR	Chicken	NR	NR	111¶	Fecal sample	NR	Both*
Ayandiran et al., 2018 (2016)	Nigeria	Cross-sectional/NR	Farm	Medium scale	Poultry	NR	NR	20	Fecal sample	Reported/GP	Both*
Ayeni et al., 2015 (2014)	Nigeria	Cross-sectional/NR	Farm	NR	Poultry	NR	NR	45	Fecal sample	NR	Phenotypic only
Badi et al., 2018 (2012)	Tunisia	Cross-sectional/NR	Farm	NR	Poultry	NR	Healthy	20	Fecal sample	NR	Both*
Belmahdi et al., 2016 (2014)	Algeria	Cross-sectional/NR	Farm	NR	Broiler	NR	Healthy	61	Cecal content	NR	Both*
ben Sallem et al., 2012 (2011)	Tunisia	Cross-sectional/NR	Farm	Intensive	Chicken	NR	NR	14	Fecal sample	NR	Both*
				Extensive	Chicken	NR	NR	8	Fecal sample	NR	Both*
Büdel et al., 2020 (2018)	Tanzania	Cross-sectional/NR	Farm	NR	Poultry	NR	Healthy	62	Fecal sample	NR	Both*
Chabou et al., 2018 (2014)	Algeria	Cross-sectional/Convenience sampling	Farm	Large scale	Broiler	NR	Healthy	503 ¶	Fecal sample	NR	Genotypic only
			Slaughterhouse	N/A	Broiler	NR	Healthy		Fecal sample	NR	Genotypic only
Chah et al., 2018 (2014-2015)	Nigeria	Cross-sectional/NR	Slaughterhouse	N/A	Broiler	NR	NR	410	Fecal sample	NR	Both*
Falgenhauer et al., 2019 (2015)	Ghana	Repeat cross-sectional	Farm	Large scale	Broiler	NR	NR	140	Fecal sample	NR	Both*
Fortini et al., 2011 -2006	Nigeria	Cross-sectional/NR	Slaughterhouse	N/A	Chicken	NR	Healthy	100	Fecal sample	NR	Genotypic only
Grami et al., 2013 (2011-2012)	Tunisia	Cross-sectional/NR	Farm	Large scale	Chicken	NR	Diseased	193	Fecal sample	NR	Both*
Hassen et al., 2020 (2018)	Tunisia	Cross-sectional/Convenience sampling	Farm	Large scale	Broiler	35 days (5 weeks)	Healthy	286	Fecal sample	Reported/DPT	Both*
Jouini et al., 2007 (NR)	Tunisia	Cross-sectional/NR	Farm	NR	Chicken	NR	NR	6	Fecal sample	NR	Both*
Katakweba et al., 2018 (2011-2013)	Tanzania	Cross-sectional/NR	Farm	Intensive	Chicken	NR	NR	97	Fecal sample	NR	Both*
				Extensive	Chicken	NR	NR	97	Fecal sample	NR	Both*
Kilani et al., 2015 (2013)	Tunisia	Cross-sectional/NR	Farm	NR	Chicken	58 weeks	Healthy	45	Fecal sample	NR	Both*
						7 weeks	Healthy	20	Fecal sample	NR	Both*
Kilani et al., 2020 (2009-2012)	Tunisia	Cross-sectional/NR	Farm	NR	Poultry	NR	Healthy	61	Fecal sample	NR	Both*
Kimera et al., 2021 (NR)	Tanzania	Cross-sectional/Convenience sampling	Farm	Small scale	Poultry	NR	NR	390	Cloacal swab	NR	Both*
Kwoji et al., 2019 (NR)	Nigeria	Cross-sectional/NR	Farm	Small scale	Broiler	NR	NR	24	Cloacal swab	NR	Phenotypic only
					Layer	NR	NR	24	Cloacal swab	NR	Phenotypic only
					Chick broiler	NR	NR	24	Cloacal swab	NR	Phenotypic only
					Pullet	NR	NR	24	Cloacal swab	NR	Phenotypic only
Langata et al., 2019 (2017)	Kenya	Cross-sectional/NR	Farm	Backyard	Broiler and layer	NR	NR	150	Fecal sample	NR	Genotypic only
Maamar et al., 2016 (2013)	Tunisia	Cross-sectional/NR	Farm	Large scale	Layer	NR	Healthy	137	Fecal sample	NR	Both*
Messaili et al., 2019 (NR)	Algeria	Cross-sectional/NR	Farm	NR	Broiler	45-47 days (6 weeks)	Healthy	100	Fecal sample	NR	Both*
Mgaya et al., 2021 (2020)	Tanzania	Cross-sectional/Purposive sampling	Slaughterhouse	N/A	Broiler	NR	NR	96	Cloacal swab	NR	Both*
					Layer	NR	NR	96	Cloacal swab	NR	Both*
Mnif et al., 2012 (2010)	Tunisia	Cross-sectional/NR	Farm	Large scale	Chicken	NR	Healthy	136	Fecal sample	NR	Both*
Moawad et al., 2018 (2016)	Egypt	Cross-sectional/NR	Farm	Large scale	Broiler	NR	Healthy	576	Cloacal swab	NR	Both*
Ojo et al., 2016 (2014)	Nigeria	Cross-sectional/NR	Farm	Small/medium sized farm	Chicken	NR	NR	143	Fecal sample	Reported/NR	Both*
				Backyard	Chicken	NR	NR	97	Fecal sample	Reported/NR	Both*
Okpara et al., 2018 (NR)				Backyard/extensive	Chicken	NR	NR	101¶	Cloacal swab	Reported/DPT & GP	Both*
				Backyard/semi-intensive	Chicken	NR	NR		Cloacal swab	Reported/DPT & GP	Both*
	Nigeria	Cross-sectional/Random sampling	Farm	Backyard/intensive	Chicken	NR	NR		Cloacal swab	Reported/DPT & GP	Both*
				Backyard/extensive	Turkey	NR	NR	7	Cloacal swab	Reported/DPT & GP	Both*
				Backyard/semi-intensive	Turkey	NR	NR	2	Cloacal swab	Reported/DPT & GP	Both*
Okubo et al., 2019 (2016-2017)	Uganda	Cross-sectional/NR	Farm	NR	Layer	NR	NR	39	Fecal sample	Reported/DPT & GP	Both*
Ramadan et al., 2018 (2015)	Egypt	Cross-sectional/NR	Farm	NR	Broiler	NR	Healthy	40	Cloacal swab	NR	Both*
Saidani et al., 2019 (2016)	Tunisia	Cross-sectional/NR	Farm	Intensive	Chicken	>30 days (4 weeks)	Healthy	258	Cloacal swab	Reported/DPT	Both*
Sghaier et al., 2019 (2013-2015)	Tunisia	Cross-sectional/NR	Farm	NR	Poultry	NR	Healthy	60	Fecal sample	NR	Both*
Vounba et al., 2019 (2011)	Senegal	Cross-sectional/Random Sampling.	Farm	NR	Chicken	NR	Healthy	50	Fecal sample	Reported/DPT	Both*

NR- Not Reported.

N/A- Not applicable.

GP – Growth promotion.

DPT – Disease prevention and/or treatment.

Both* - Both genotypic and phenotypic resistance detection has been carried out.

¶ - Number of samples per setting has not been specified.

#### 3.2.3 Study setting and production systems

The majority of the studies were carried out at farm level (29 studies, 87.9%), three (Fortini et al., 2011; Chah et al., 2018; Mgaya et al., 2021) were carried out at slaughterhouse level while one of the studies (Chabou et al., 2018) was conducted at both farm and slaughterhouse levels. At farm level, only seven studies (24.1%) provided details of ABU on the farm for disease prevention and/or treatment (Saidani et al., 2019; Vounba et al., 2019; Hassen et al., 2020), growth promotion (Ayandiran et al., 2018) or both purposes (Okpara et al., 2018; Okubo et al., 2019) (**Table 2**), whilst one study (Ojo et al., 2016) did not report the reason for ABU. The antibiotics used include one or more fluoroquinolones (enrofloxacin, ofloxacin, norfloxacin ciprofloxacin, flumequine); tetracyclines (oxytetracycline, doxycycline, tetracycline); macrolides (erythromycin); penicillins (benzylpenicillin, amoxicillin, amoxicillin/clavulanic acid, ampicillin); aminoglycosides (gentamicin, streptomycin, neomycin); phenicols (chloramphenicol, florfenicol); polymyxins (colistin); sulfonamides (trimethoprim/sulphamethoxazole, sulphonamide); nitrofurans (furazolidone). Tetracyclines were the most frequently reported antimicrobial agents, in six out of the seven studies (**Table 3**).

**Table 3:** Antibiotic use Reported in eligible studies.

Publication details	Tetracyclines			Fluoroquinolones									Macrolides	Penicillins				Aminoglycosides			Phenicols		Polymyxins	Sulfonamides		Nitrofurans
	OXY	DOX	TET	ENR		OFX		NOR		CIP		FLU	ERY	BENPEN	AMX	AMC	AMP	GEN	STR	NEO	CHL	FFN	CST	STX	SMZ	FUR
Ayandiran et al., 2018	+			+																						
Hassen et al., 2020		+				+																+				
Ojo et al., 2016			+	+													+	+	+						+	
Okpara et al., 2018	+	+		+				+		+		+	+		+	+	+	+	+	+	+		+	+	+	+
Okubo et al., 2019	+													+					+					+	+	
Saidani et al., 2019*	+			+											+						+		+			
Vounba et al., 2019			+	+				+																		

+use reported.

*Details of individual antibiotic use not provided in this study.

OXY, oxytetracycline; DOX, doxycycline; TET, tetracycline; ENR, enrofloxacin; OFX, ofloxacin; NOR, norfloxacin; CIP, ciprofloxacin; FLU, flumequine; ERY, Erythromycin; BENPEN, benzylpenicillin; AMX, amoxicillin; AMC, amoxycillin/clavulanic acid; AMP, ampicillin; GEN, gentamicin; STR, streptomycin; NEO, neomycin; CHL, chloramphenicol; FFN, florfenicol; STX, trimethoprim/sulphamethoxazole; SMZ, sulphonamide; CST, colistin; FUR, furazolidone.

Nineteen (57.6%) of the eligible studies provided details of the type of production systems. Seven (22.6%) studies were conducted on large-scale farms, three were in small and medium scale farms, and two in backyard farms. In one study, birds were sampled from small and medium- sized commercial farms and backyard farms. ESBL-*E. coli* levels were generally higher in large-scale farms (23.8% - 93.8%) than in small and medium-scale farms (0% - 35.3%). All three studies conducted in backyard production systems (Ojo et al., 2016; Okpara et al., 2018; Langata et al., 2019) reported low ESBL levels ranging from 0 to 2.9%. Two studies (ben Sallem et al., 2012; Mgaya et al., 2021) assessed ESBL-*E. coli* in both intensive and extensive systems while one study (Saidani et al., 2019) was carried out on a farm with an intensive production system. However, not all reported ESBL proportions to allow for comparisons (**Table 2**).

#### 3.2.4 Study population

Most of the studies sampled chickens (26 studies, 78.8%). Out of these, eight (30.8%) assessed ESBLs in broilers, two (7.7%) in layers, and three (11.5%) in both broilers and layers. One study (3.8%) sampled both chickens and turkeys (Okpara et al., 2018). The age of the birds sampled was reported in only five studies (15.2%) (Kilani et al., 2015; Agabou et al., 2016; Messaili et al., 2019; Saidani et al., 2019; Hassen et al., 2020), and it ranged from four to 58 weeks. Sixteen (48%) of the studies were carried out in healthy birds, one study (Grami et al., 2013) was carried out in diseased birds and the remaining 16 studies did not report the health status of sampled birds. The studies assessed for the presence of ESBLs in fecal samples (25 studies, 75.8%), cloacal swabs (7 studies) and cecal content (1 study) (**Table 2**).

Twenty-eight studies (84.8%) employed both phenotypic and genotypic methods to assess ESBLs, three studies (9.1%) employed only genotypic methods, and two studies (6.1%) employed only phenotypic methods to assess for the presence of ESBLs.

ESBL-producing *E. coli* were detected both at farm and slaughterhouse level in all countries apart from Uganda. The gene families isolated by the studies included in this review were *bla*_TEM_, *bla*_CTX-M_, *bla*_OXA_, and *bla*_SHV_. Resistance among ESBL-*E. coli* was reported in 24 studies (72.7%). Out of these, 12 (50%) reported the presence of Multidrug-Resistant (MDR) isolates (that presented resistance to at least three antibiotic classes) (Magiorakos et al., 2012).

### 3.3 Frequency of ESBL-producing *E. coli* and diversity of ESBL genes by region

#### 3.3.1 North Africa

Seventeen studies from North Africa assessed ESBLs in *E. coli*. The studies were conducted in three countries namely Algeria, Egypt, and Tunisia, and most of the studies (11 studies, 64.7%) were carried out in Tunisia. All studies obtained samples at farm level. One study (Chabou et al., 2018) obtained samples from farms and slaughterhouses. The majority (12 studies, 70.6%) were published after 2015. Sixteen studies (94.1%) employed phenotypic methods to assess for ESBLs. Out of these, 12 studies (75%) reported ESBL production. In three studies (Jouini et al., 2007; Agabou et al., 2016; Badi et al., 2018), no ESBL-producing *E. coli* were isolated. In one study, the phenotypic count was not reported. The highest proportion of ESBL-*E. coli* (98%) in this region was reported in chickens raised intensively in Tunisia (Saidani et al., 2019) (**Table 4**).

**Table 4:** ESBL proportions, gene diversity, and resistance patterns by region.

S/N	Publication Details	Location	Phenotypic ESBL count (proportion %)	Genotypic ESBL count (Proportion %)	ESBL Genes detected (count)	Phenotypic Antibiotic Resistance patterns
NORTH AFRICA						
1.	Agabou et al., 2016	Algeria	0/70 (0 %)	0/36 (0 %)	None.	N/A
2.	Belmahdi et al., 2016	Algeria	16/20 (80%)	20/20 (100%)	CTX-M-1 (2) * TEM-1 (20) * SHV-12 (14) *	Fluoroquinolones, Aminoglycosides, Penicillins, Monobactams, cephalosporins
3.	Chabou et al., 2018	Algeria	N/A	NR	CTX-M (46) TEM (128) SHV (83) OXA-58 (132)	NR
4.	Messaili et al., 2019	Algeria	1/100 (1%)	1/100 (1%)	CTX-M-1 (1)	Tetracyclines, Sulphonamides, Fluoroquinolones, Aminoglycosides, Penicillins, cephalosporins ¶
5.	Moawad et al., 2018	Egypt	15/63 (23.8%)	15/63 (23.8%)	CTX-M-15 (1) * TEM (13) * SHV (1) * OXA-1 (1); OXA-7 (2) *	Sulphonamides, Fluoroquinolones, Aminoglycosides, Penicillins, Monobactams, Polymyxins, cephalosporins ¶
6.	Ramadan et al., 2018	Egypt	NR	2	TEM (2)	NR
7.	Badi et al., 2018	Tunisia	0/13 (0%)	0	None	N/A
8.	Ben Sallem et al., 2012	Tunisia	8/10 (80%)	8/10 (80%)	CTX-M-1 (8) * TEM-1B (1) * TEM-135 (1) *	Tetracyclines, Sulphonamides, Fluoroquinolones, Aminoglycosides
9.	Grami et al., 2013	Tunisia	8	8	CTX-M-1 (7); CTX-M- 9 (1)	Tetracyclines, Sulphonamides, Fluoroquinolones, Aminoglycosides
10.	Hassen et al., 2020	Tunisia	60/64 (93.8%)	60/60 (100%)	CTX-M-1 (41); CTX- M-14 (1); CTX-M-55 (18) *	Tetracyclines, Sulphonamides, Fluoroquinolones, Phenicols ¶
					TEM-1B (55) *	
11.	Jouini et al., 2007	Tunisia	0	0	None	N/A
12.	Kilani et al., 2015 ^a^	Tunisia	11	11	CTX-M-1 (11) * TEM-1 (1) *	Tetracyclines, Sulphonamides, Fluoroquinolones, Aminoglycosides
	Kilani et al., 2015 ^b^		6	5	CTX-M-1 (5)	
13.	Kilani et al., 2020	Tunisia	1	1	CTX-M-1 (1)	Tetracyclines, Sulphonamides, Fluoroquinolones, Aminoglycosides, Penicillins ¶
14..	Maamar et al., 2016	Tunisia	35/48 (72.9%)	35/48 (72.9%)	CTX-M-1 (29); CTX- M-14 (1); CTX-M-15 (5) * TEM-1 (8) *	Tetracyclines, Sulphonamides, Fluoroquinolones, Aminoglycosides, Phenicols
15.	Mnif et al., 2012	Tunisia	43/67 (64.2%)	43/67 (64.2%)	CTX-M-1 (39); CTX- M-15 (4) * TEM-1 (26) *	Tetracyclines, Sulphonamides, Fluoroquinolones, Aminoglycosides ¶
16.	Saidani et al., 2019	Tunisia	49/50 (98%)	49/50 (98%)	CTX-M-1 (35); CTX- M-15 (3); CTX-M-55 (6) SHV-12 (6)	Tetracyclines, Sulphonamides, Fluoroquinolones, Aminoglycosides, Phenicols, Polymyxins.
17.	Sghaier et al., 2019	Tunisia	31	31/31 (100%)	CTX-M-1 (29); CTX- M-15 (2)	Tetracyclines, Sulphonamides, Fluoroquinolones, Aminoglycosides
WEST AFRICA						
1.	Falgenhauer et al., 2019	Ghana	45	45	CTX-M-15 (43); CTX- M-15 (3) SHV-12 (2)	NR
2.	Ayeni et al., 2015	Nigeria	1/20 (5%)	N/A	N/A	Penicillins, cephalosporins
3.	Aworh et al., 2020	Nigeria	14/22 (63.6%)	2/22 (9.1%)	CTX-M-15 (1); CTX- M-65 (1)	Tetracyclines, Sulphonamides, Fluoroquinolones, Aminoglycosides, Phenicols, Penicillins, Macrolides, cephalosporins ¶
4.	Ayandiran et al., 2018	Nigeria	3/52 (5.8%)	3/3 (100%)	CTX-M-15 (3) * TEM (3) *	Tetracyclines, Fluoroquinolones, Aminoglycosides
5.	Chah et al., 2018	Nigeria	9/10 (90%)	9/9 (100%)	CTX-M-1 (3); CTX-M- 15 (5) * TEM-1 (1) *	Tetracyclines, Sulphonamides, Fluoroquinolones, Aminoglycosides, Phenicols, Penicillins, cephalosporins ¶
6.	Fortini et al., 2011	Nigeria	N/A	15/96 (15.6%)	CTX-M-15 (1) * TEM-1 (15) *	N/A
7.	Kwoji et al., 2019 ^c^	Nigeria	6/17 (35.3%)	N/A	N/A	Monobactams, cephalosporins
	Kwoji et al., 2019 ^d^		5/13 (38.5%)	N/A	N/A	
	Kwoji et al., 2019 ^e^		6/20 (30%)	N/A	N/A	
	Kwoji et al., 2019 ^f^		4/15 (26.7%)	N/A	N/A	
8.	Ojo et al., 2016 ^g^	Nigeria	4/143 (2.8%)	4/143 (2.8%)	CTX-M-15 (4)	Tetracyclines, Sulphonamides, Fluoroquinolones, Aminoglycosides, Phenicols, Carbapenems, cephalosporins
	Ojo et al., 2016 ^h^		0/97 (0%)	0	None	
9.	Okpara et al., 2018 ^i^	Nigeria	3/101 (2.9%)	3/101 (2.9%)	CTX-M-1 (1); CTX-M- 15 (1); CTX-M-27 (1)	Tetracyclines, Sulphonamides, Fluoroquinolones, Aminoglycosides, Phenicols ¶
	Okpara et al., 2018 ^j^		1/9 (11.1%)	1/9 (11.1%)	CTX-M-15 (1)	
10.	Vounba et al., 2019	Senegal	NR	19	CTX-M (2) TEM (17)	Tetracyclines, Sulphonamides, Fluoroquinolones, Aminoglycosides ¶
EAST AFRICA						
1.	Langata et al., 2019	Kenya	N/A	11	TEM (11)	N/A
2.	Büdel et al., 2020	Tanzania	NR	NR	CTX-M-9 ; CTX-M-15	Tetracyclines, Sulphonamides, Fluoroquinolones, Aminoglycosides, Penicillins, Glycylclines, polymymins, carbapenems, cephalosporins
3.	Katakweba et al., 2018 ^k^	Tanzania	32/32 (100%)	32/32 (100%)	CTX-M (32)	Tetracyclines, Sulphonamides, Fluoroquinolones, Aminoglycosides, Penicillins, cephalosporins ¶
	Katakweba et al., 2018 ^l^		22/22 (100%)	22/22 (100%)	CTX-M (22)	
4.	Kimera et al., 2021	Tanzania	NR	7	CTX-M (7)	Tetracyclines, Sulphonamides, Fluoroquinolones, Aminoglycosides, Phenicols, Penicillins, carbapenems, cephalosporins ¶
5.	Mgaya et al., 2021 ^c^	Tanzania	1	0	None	Tetracyclines, Sulphonamides, Fluoroquinolones, Penicillins, Carbapenems, cephalosporins ¶
	Mgaya et al., 2021 ^d^		4/4 (100%)	1/4 (25%)	CTX-M (1)	
6.	Okubo et al., 2019	Uganda	0/63 (0%)	0	None	N/A

* co-existence of genes observed in isolates in this study

¶ Study reported MDR ESBL E. coli isolates

a- 58 weeks, b- 7 weeks, c- broiler, d- layer, e- chick broiler, f- pullet, g- small/medium scale, h-backyard farm, i-chicken, j-turkey, k- intensive, l- extensive

NR- Not reported N/A – Not applicable

**Figure 4:**
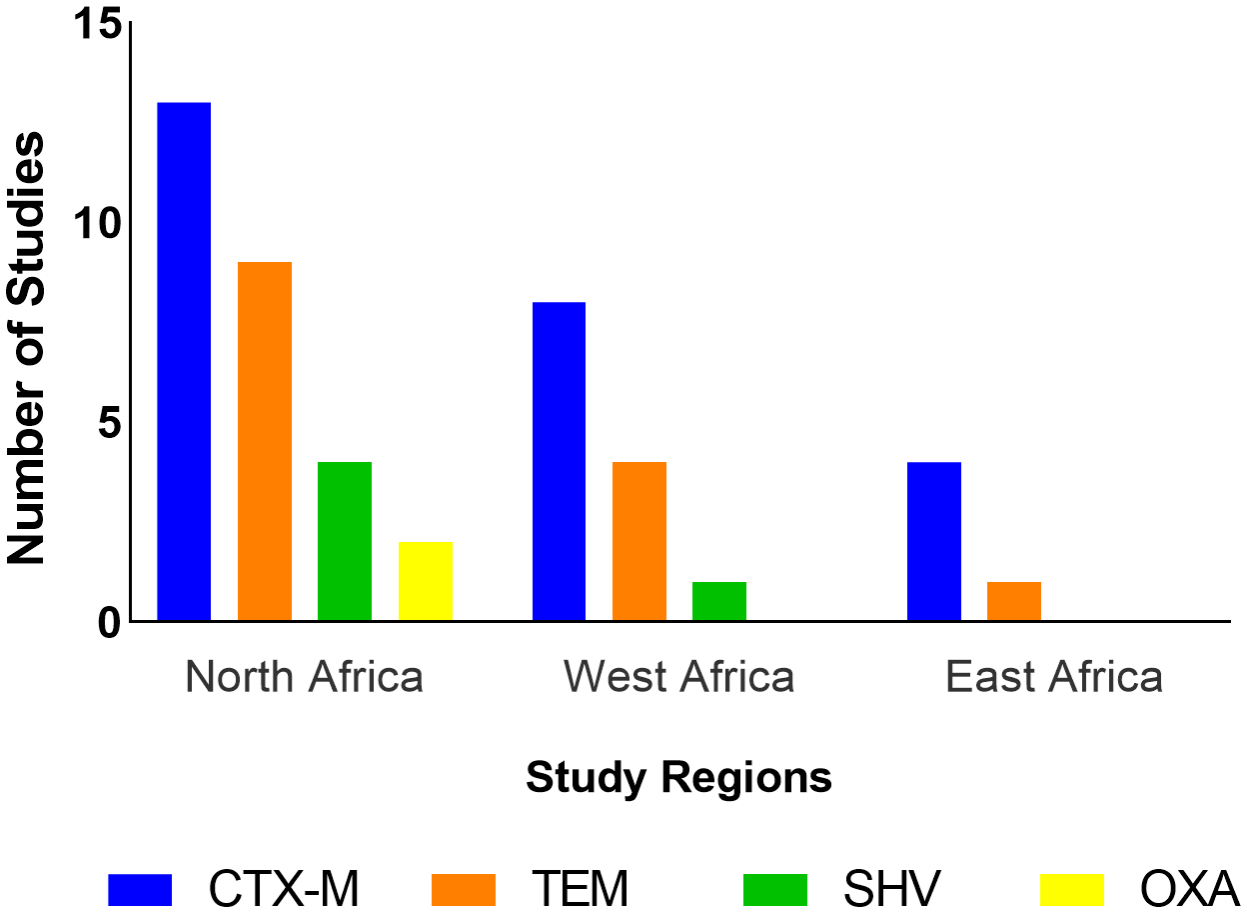
Frequency of studies reporting detection of ESBL gene families by region.

All 17 studies from North Africa used genotypic methods. Out of these, 14 (82.4%) detected ESBL genes. The ESBL genes detected in this region belonged to the CTX-M, TEM, SHV, and OXA families (**Figure 4**). CTX-M-1 was the most frequently isolated gene in this region, being detected in 11 studies (64.7%) followed by CTX-M-15 (5 studies, 29.4%), TEM-1 (4 studies, 23.5%) CTX-M-55 (2 studies, 11.8%), and SHV-12 (2 studies, 11.8%). Coexpression of genes was reported in seven studies (41.2%) in this region (**Table 4**).

**Figure 5:**
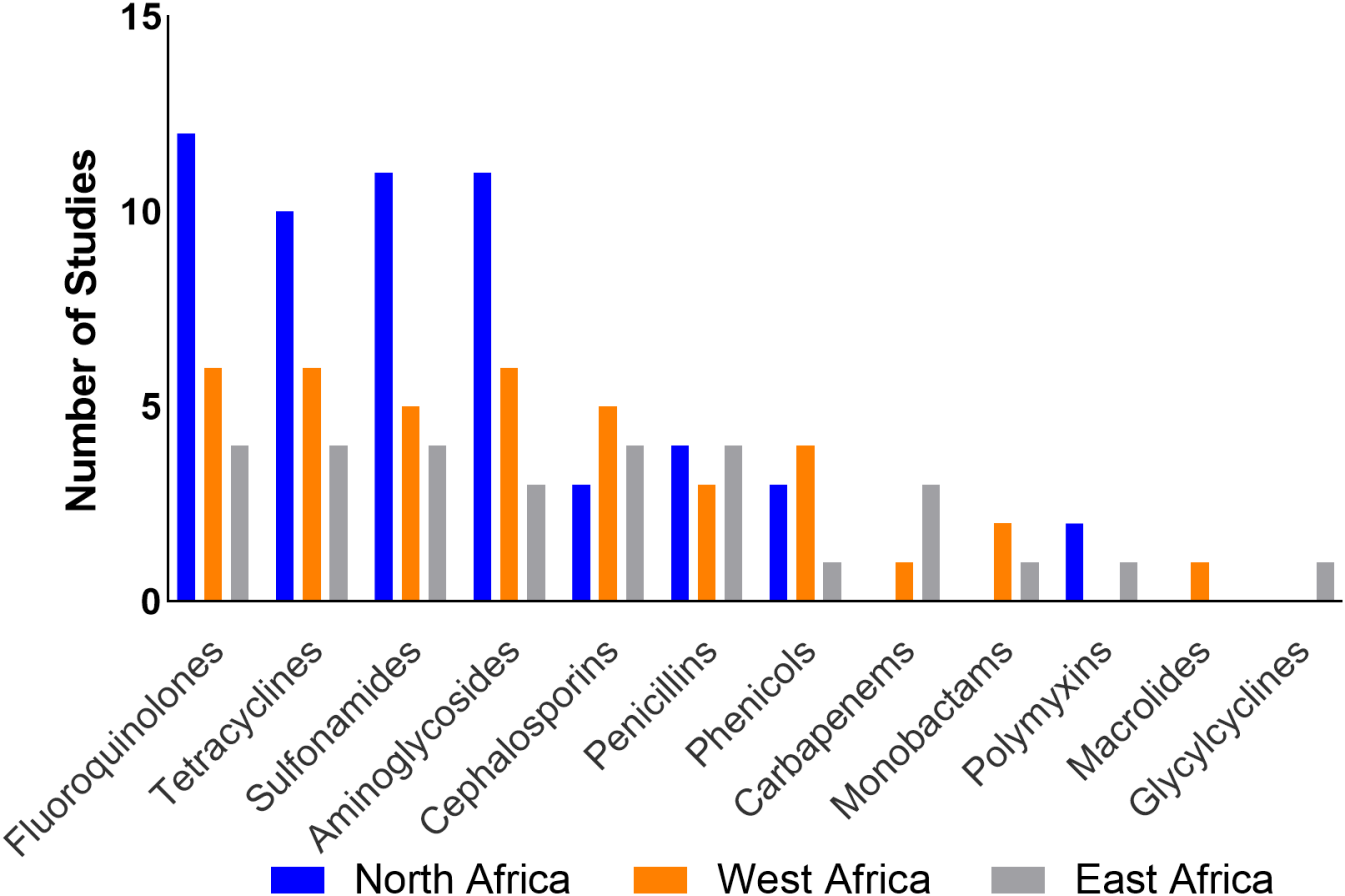
Frequency of studies reporting AMR patterns by region.

Only two studies (11.8%) from Tunisia (Saidani et al., 2019; Hassen et al., 2020) reported use of polymyxins, tetracyclines, quinolones, penicillins, and phenicols in poultry. ESBL-producing *E. coli* in this region presented resistance to one or more antibiotics across eight classes (Aminoglycosides, cephalosporins, fluoroquinolones, monobactams, penicillins, polymyxins, sulphonamides, and tetracyclines). Fluoroquinolones represent the antibiotic class for which resistance was most often reported (**Table 4**; **Figure 5**). Five studies (Mnif et al., 2012; Moawad et al., 2018; Messaili et al., 2019; Hassen et al., 2020; Kilani et al., 2020) reported the presence of MDR ESBL *E. coli* isolates.

#### 3.3.2 West Africa

Ten studies were conducted in Ghana, Nigeria, and Senegal. Most of the studies (8 studies, 80%) were from Nigeria. Eight studies sampled poultry at farm level while two studies (20%) from Nigeria (Fortini et al., 2011; Chah et al., 2018) were conducted at slaughterhouse level. Eight of nine studies that used phenotypic methods reported ESBL production. In one study, the phenotypic count was not reported. The highest proportion of ESBL-producing *E. coli* (90%) in this region was reported in Nigeria in a study that sampled broilers at slaughterhouse level (Chah et al., 2018) (**Table 4**).

Eight studies employed genotypic methods to assess ESBL-*E. coli*; ESBL genes were detected in all studies. Only genes from the TEM, CTX-M, and SHV families were isolated (**Figure 4**). CTX-M-15 was the most isolated gene, being detected in seven (70%) of the studies followed by TEM-1 (2 studies, 20%). Coexpression of genes has also been reported in three studies (30%) in this region (**Table 4**).

Four studies (40%) reported ABU (Ojo et al., 2016; Ayandiran et al., 2018; Okpara et al., 2018; Vounba et al., 2019); the most commonly used antimicrobials belonged to the tetracyclines, fluoroquinolones, macrolides, penicillins, aminoglycosides, phenicols, polymyxins, sulfonamides and nitrofurans classes. Most of the studies reported resistance of ESBLs to one or more tetracyclines, fluoroquinolones, and aminoglycosides. Four studies (Chah et al., 2018; Okpara et al., 2018; Vounba et al., 2019; Aworh et al., 2020) reported the presence of MDR ESBL *E. coli* isolates (**Table 4**; **Figure 5**).

#### 3.3.3 East Africa

Six studies from Kenya, Tanzania, and Uganda were identified. Tanzania contributed the highest number of studies (4 studies, 66.7%). Five studies (83.3%) were conducted at farm level; only one study (16.7%) (Mgaya et al., 2021) was conducted at slaughterhouse level. Five studies assessed ESBLs using phenotypic methods; ESBL production was reported in two studies (33.3%) (Katakweba et al., 2018; Mgaya et al., 2021) at 100% level. Both studies sampled chickens in Tanzania; one in a slaughterhouse (Chabou et al., 2018) and the other in a commercial farm (Büdel et al., 2020). In two studies, the phenotypic counts were not reported. One study (Okubo et al., 2019) reported 0% ESBLs at farm level. This was the only study carried out in Uganda (**Table 4**).

Only genes from the family CTX-M and TEM were reported in this region (**Figure 4**). Gene variants reported include CTX-M-9 and CTX-M-15; both were detected in one study (Büdel et al., 2020) (**Table 4**).

Only one study, which sampled chickens in subsistence and commercial farms, reported the use of antibiotics which included aminoglycosides, fluoroquinolones, macrolides, penicillins, penicillin-streptomycin combination, sulfonamides, sulfonamide-trimethoprim combination and tetracyclines (Okubo et al., 2019) but no ESBL-*E. coli* isolates were detected in this study. Four studies however reported ESBL-*E. coli* resistance to tetracyclines, sulphonamides, fluoroquinolones, penicillins, and cephalosporins. Three studies reported presence of MDR ESBL *E. coli* isolates (**Table 4**; **Figure 5**). 

### 3.4 ESBL detection methods

Most studies 28 studies, 84.8%) employed a combination of both phenotypic and genotypic tests. Two studies (6.1%) conducted in Nigeria (Ayeni et al., 2015; Kwoji et al., 2019) used only phenotypic methods. Three studies (9.1%) (Fortini et al., 2011; Chabou et al., 2018; Langata et al., 2019) used only genotypic methods (**Table 5**).

**Table 5:** Methods and guidelines employed for ESBL detection.

Publication Details	Location/Region	Antibiotic susceptibility test	Phenotypic confirmatory test	Genotypic detection test	Screened Genes	Accreditation body
Agabou et al., 2016	Algeria/North Africa	Disk diffusion	Double disk synergy test	PCR	TEM, CTX-M, SHV	EUCAST
Belmahdi et al., 2016	Algeria/North Africa	Disk diffusion	Double disk synergy test	PCR	TEM, CTX-M, SHV	CA-SFM
Chabou et al., 2018	Algeria/North Africa	N/A	N/A	PCR	TEM, CTX-M, SHV	N/A
Messaili et al., 2019	Algeria/North Africa	Disk diffusion	Double disk synergy test	PCR	TEM, CTX-M, SHV	CA-SFM
Moawad et al., 2018	Egypt/North Africa	Broth microdilution	VITEK test	Microarray analysis	TEM, CTX-M, OXA, SHV	German Institute for Standardization
Ramadan et al., 2018	Egypt/North Africa	Broth microdilution	Broth microdilution	PCR	TEM, CTX-M, OXA, SHV	CLSI
Badi et al., 2018	Tunisia/North Africa	Disk diffusion	Double disk synergy test	PCR	TEM, CTX-M, SHV	CLSI
ben Sallem et al., 2012	Tunisia/North Africa	Disk diffusion	Double disk synergy test	PCR	TEM, CTX-M, OXA, SHV	CLSI
Grami et al., 2013	Tunisia/North Africa	Disk diffusion	Double disk synergy test	PCR	CTX-M	CA-SFM
Hassen et al., 2020	Tunisia/North Africa	Disk diffusion	Double disk synergy test	PCR	TEM, CTX-M, SHV	CLSI
Jouini et al., 2007	Tunisia/North Africa	Disk diffusion	Double disk synergy test	PCR	TEM, CTX-M, SHV	CLSI
Kilani et al., 2015	Tunisia/North Africa	Disk diffusion	Double disk synergy test	PCR	TEM, CTX-M, SHV	CLSI
Kilani et al., 2020	Tunisia/North Africa	Disk diffusion	Double disk synergy test	PCR	TEM, CTX-M, SHV	CLSI
Maamar et al., 2016	Tunisia/North Africa	Disk diffusion	Double disk synergy test	PCR	TEM, CTX-M, OXA, SHV	CLSI
Mnif et al., 2012	Tunisia/North Africa	Disk diffusion	Double disk synergy test	PCR	TEM, CTX-M, OXA, SHV	CLSI
Saidani et al., 2019	Tunisia/North Africa	Disk diffusion	Double disk synergy test	PCR	CTX-M	CA-SFM
Sghaier et al., 2019	Tunisia/North Africa	Disk diffusion	Double disk synergy test	PCR	TEM, CTX-M, OXA, SHV	CLSI
Falgenhauer et al., 2019	Ghana/West Africa	NR	Combination disc test	PCR	Not specified	EUCAST
Ayeni et al., 2015	Nigeria/West Africa	Disk diffusion	Double disk synergy test	PCR	N/A	CLSI
Aworh et al., 2020	Nigeria/West Africa	Disk diffusion	NR	PCR	TEM, CTX-M, OXA	CLSI
Ayandiran et al., 2018	Nigeria/West Africa	Disk diffusion	VITEK test	PCR	Not specified	CLSI
Chah et al., 2018	Nigeria/West Africa	Disk diffusion	Combination disc test	PCR	TEM, CTX-M, OXA, SHV	CLSI
Fortini et al., 2011	Nigeria/West Africa	N/A	N/A	PCR	TEM, CTX-M, SHV	N/A
Kwoji et al., 2019	Nigeria/West Africa	Disk diffusion	Combination disc test	PCR	N/A	CLSI
Ojo et al., 2016	Nigeria/West Africa	Disk diffusion, broth microdilution and broth macrodilution	Double disk synergy test	PCR	TEM, CTX-M, SHV	CLSI
Okpara et al., 2018	Nigeria/West Africa	Disk diffusion	Combination disc test	PCR	CTX-M	CLSI
Vounba et al., 2019	Senegal/West Africa	Disk diffusion	Double disk synergy test	PCR	TEM, CTX-M, OXA, SHV	CLSI
Langata et al., 2019	Kenya/East Africa	N/A	N/A	PCR	TEM, CTX-M, OXA, SHV	N/A
Büdel et al., 2020	Tanzania/East Africa	Broth microdilution	NR	Microarray analysis	CTX-M	EUCAST
Katakweba et al., 2018	Tanzania/East Africa	Disk diffusion	VITEK test	PCR	CTX-M	CLSI
Kimera et al., 2021	Tanzania/East Africa	Disk diffusion	Combination disc test	PCR	TEM, CTX-M, SHV	CLSI
Mgaya et al., 2021	Tanzania/East Africa	Disk diffusion	Combination disc test	PCR	TEM, CTX-M, SHV	CLSI
Okubo et al., 2019	Uganda/East Africa	Broth microdilution	NR	PCR	TEM, SHV, OXA	CLSI/EUCAST

N/A, Not Applicable; N/R, Not Reported.

*E. coli* isolates were assessed for susceptibility to over 40 antibiotics, including 3rd generation cephalosporins, across all studies. The majority of the studies (24 studies, 72.7%) used the disk diffusion test, whilst four studies used broth microdilution, and one study used a combination of disk diffusion, broth microdilution, and broth macrodilution tests (Ojo et al., 2016). In one study (Falgenhauer et al., 2019), the details of the antimicrobial susceptibility testing (AST) were not provided. Phenotypic confirmatory tests were carried out across studies using the double-disk synergy test (DDST) (17 studies), the combination disc test (6 studies), BioMérieux Inc’s VITEK test (an automated bacterial identification and susceptibility testing system) (3 studies), and broth microdilution (1 study). In three studies (Okubo et al., 2019; Aworh et al., 2020; Büdel et al., 2020), phenotypic confirmatory tests were not reported. Instead, these studies employed genotypic confirmatory methods (**Table 5**).

Genotypic detection of ESBL genes (*bla*_TEM_, *bla*_CTX-M_, *bla*_OXA_, and *bla*_SHV_) was carried out using Polymerase Chain Reaction (PCR) and sequencing in most of the studies (29 studies, 93.5%). Microarray analysis has been employed in only two studies (Moawad et al., 2018; Büdel et al., 2020). Fifteen studies (45.5%) screened for three gene families, nine studies screened for four gene families, five screened for only the CTX-M gene family and two studies (Ayandiran et al., 2018; Falgenhauer et al., 2019) did not specify the panel of genes screened.

### 3.5 Guidelines adopted for AST by the studies

All studies reported the guidelines followed when carrying out AST. Most of the studies (21 studies, 70%) followed the guidelines from the Clinical and Laboratory Standards Institute (CLSI). Three studies (Agabou et al., 2016; Falgenhauer et al., 2019; Büdel et al., 2020) followed guidelines from European Committee on Antimicrobial Susceptibility Testing (EUCAST), four studies (Grami et al., 2013; Belmahdi et al., 2016; Messaili et al., 2019; Saidani et al., 2019) followed guidelines from Antibiogram Committee of the French Society for Microbiology (CA-SFM), one study (Moawad et al., 2018) adopted guidelines from the German Institute for Standardization while one (Okubo et al., 2019) adopted the joint CLSI/EUCAST guidelines (**Table 5**).

## 4 Discussion

This review aimed to determine the frequency of ESBL-producing *E. coli* in poultry in Africa at farm and slaughterhouse levels. Due to the heterogeneity of studies, arising from various methods of assessing and reporting phenotypic and genotypic proportions of ESBLs, a meta-analysis could not be carried out to estimate overall levels of ESBL-*E. coli* in Africa. This review identified 33 studies that assessed ESBL-producing *E. coli* isolates in poultry at farm and slaughterhouse levels across Algeria, Egypt, Ghana, Kenya, Nigeria, Senegal, Tanzania, Tunisia, Uganda. Twenty-nine (87.9%) of the eligible studies confirmed ESBL production through phenotypic detection, genotypic detection, or both. ESBL genes from four families namely: TEM, CTX-M, SHV, and OXA were detected in poultry in the eligible studies included in this review. CTX-M genes were the most frequently isolated.

The levels of ESBLs in poultry varied greatly across studies, ranging from 0% in Algeria, Tunisia, and Uganda to 100% in Tanzania. This can probably be explained by variations in resistance detection methods, types of samples obtained, sampling periods, geographical locations, types of production systems and animal husbandry practices. Most of the findings were based on convenience or unspecified sampling strategies, making it difficult to arrive at generalized conclusions on the levels of ESBLs in poultry populations in these countries. In one study carried out in backyard farms in Nigeria, a prevalence of ESBL-*E. coli* at 2.97% was estimated (Okpara et al., 2018). Nonetheless, it would be impractical to make a justifiable inference based on only one study. Notably, this level is significantly lower than the levels of ESBL-*E. coli* in poultry reported in studies from Europe and Asia which range from 13.7% to 100% (Dierikx et al., 2012; Huijbers et al., 2014; Blaak et al., 2015; Umair et al., 2019). It is also lower than the ESBL levels of *E. coli* reported in other food-producing animals (Olowe et al., 2015; Braun et al., 2016) and human populations (Kiiru et al., 2012; Tufa et al., 2020) in Africa.

Most studies identified in this review were conducted in the North Africa region; overall, Tunisia was the country with the highest number of studies. This review also identified Nigeria and Tanzania as the countries with the highest number of studies in West and East Africa respectively. The frequent reporting of ESBLs in these countries does not necessarily equate to a high burden. Rather, it can be argued that resistance due to ESBL has been recognized as an urgent public health problem in these countries (Musa et al., 2020) hence, the increase in the number of publications.

CTX-M genes were most frequently isolated in eligible studies with CTX-M-1 and CTX-M-15 variants being the most isolated in North and West Africa respectively. This frequency of detection of CTX-M is in agreement with the findings of studies conducted in poultry populations in Europe and Asia (Ewers et al., 2012; Gundran et al., 2019). The TEM, SHV, and OXA genes have also been detected but the OXA gene was the least isolated, being detected only in North Africa. This is probably because OXA is mostly isolated in *Pseudomonas aeruginosa* (Poirel et al., 2010; Potron et al., 2015) and this review focused on ESBLs in *E. coli*. A study reported a high prevalence of MDR *P. aeruginosa* in humans in countries in the North Africa region (Al-Orphaly et al., 2021). The detection of *bla*_OXA_ in *E. coli* isolates from poultry in this region is not unexpected as horizontal gene transfer of ESBLs between different bacteria species is well documented (Bajpai et al., 2017; Horcajada et al., 2019). Nonetheless, only one-third of the studies included in this review screened for the OXA gene so underreporting of this gene cannot be ruled out. OXA-58 was detected in a study conducted in large scale intensive farms and slaughterhouses in Algeria (Chabou et al., 2018) and this gene has been linked to the expression of phenotypic resistance to carbapenems (Saino et al., 2015). Unfortunately, phenotypic resistance patterns were not reported in this study.

The coexistence of ESBL genes was reported in ten studies (30.3%) and in nine of these, *bla*_TEM_ co-existed with *bla*_CTX-M_ within the same ESBL-*E. coli* strains. This is corroborated by the findings of Jena et al. (2017) and Sharma et al. (2013) that reported that CTX-M/TEM coexistence is the most commonly observed.

Major concerns have been raised about the risk of zoonotic transfer of ESBLs from poultry to humans in Africa by several investigators. This is mainly based on the presence of the same CTX-M ESBL gene variants in poultry and humans (Alonso et al., 2017; Falgenhauer et al., 2019; Aworh et al., 2020). The practice of using poultry litter as soil fertilizers for crop production (Adeleye et al., 2010) and the detection of some genetically related ESBL-*E. coli* isolates in poultry and poultry farm environments (Tansawai et al., 2019) highlight a possible spread of ESBL-*E. coli* from poultry to humans. This constitutes a risk to poultry farmers and farm attendants through contact with the contaminated animal-related environment and consumers through consumption of possibly contaminated poultry meat and crops. On the other hand, ESBL gene variants such as *bla*_CTX-M-15_ and *bla*_TEM-1_ which are also commonly associated with clinical and community settings within and outside Africa (Zhao and Hu, 2013; Storberg, 2014; Mshana et al., 2016) were detected in poultry isolates across studies from the three regions included in this review. This suggests the possibility of a zoonotic spread in the opposite direction, humans to poultry, or the exposure of both human and poultry populations to a common environmental source. Notably, the role of human sewage in the contamination of the environment with ESBL bacteria has been postulated within and outside Africa (Benavides et al., 2018; Berendes et al., 2020).

Although research confirming the direction of spread of ESBLs is sparse, the circulation of ESBLs between poultry, humans, and the environment is highly probable. The persistence of ESBLs can be linked to the dispersion of ESBL-producing bacterial clones, exchange of genes that encode ESBLs, or transfer of gene-carrying plasmids. The variety of these mechanisms make the identification of the sources and transmission routes of ESBL bacteria difficult (Valentin et al., 2014).

The combination disc test (a test which measures the inhibition zone around a disk of cephalosporin and around a disk of the same cephalosporin plus clavulanate) or the E test (which quantifies the synergy between extended-spectrum cephalosporins and clavulanate) are the recommended confirmatory phenotypic tests by the CLSI and EUCAST guidelines (Soliman et al., 2020; EUCAST, 2021). However, only six studies (20%) which assessed phenotypic resistance employed the combination disc method, and no study used the E test. Seventeen studies (56.7%) used the DDST, and the rest of the studies used the VITEK test or broth microdilution. While it is simple and easy to interpret, a reduced sensitivity of the DDST (ranging from 79% - 97%) has been reported (EFSA, 2011; Giriyapur et al., 2011). With this test, false negatives can occur for isolates harbouring SHV-2, SHV-3, and TEM-12 genes (Rawat and Nair, 2010), none of which were identified by studies included in this review. It is also worth noting that DDST was employed in three out of the four studies that reported 0% ESBL genes in *E. coli* isolates. Fortunately, most of the studies also carried out genotypic confirmation using PCR or microarray analysis. However, the common practice among studies was to carry out genotypic screening using isolates of ESBL producers previously confirmed by phenotypic tests. This could have led to an underestimation of ESBL levels especially in studies where genotypic confirmation was not carried out.

Resistance to one or more antimicrobial classes, such as aminoglycosides, fluoroquinolones, sulphonamides and tetracyclines, was the most frequently reported in studies across the three regions. Additionally, MDR ESBL-*E. coli* isolates were reported in 12 studies (36.4%). This is not unexpected because the plasmids on which ESBLs are located often carry resistance genes to other antimicrobial classes (Bajpai et al., 2017). Research suggests an association between resistance to quinolones and ESBL production which can be explained by the coexistence of ESBL genes with *qnr* genes which code for resistance to quinolones (Paterson and Bonomo, 2005; Pakzad et al., 2011). This supports the findings of this review. Although only seven studies (21%) reported data on ABU, it is interesting to note that these studies reported the use of all the four antibiotic classes (aminoglycosides, fluoroquinolones, sulphonamides and tetracyclines) to which resistance was observed across the three regions. The resistance patterns of ESBL-*E. coli* isolates to commonly used antibiotics in this study may add to the evidence which implicates ABU as a risk factor for the development of AMR (Depoorter et al., 2012).

Although not all studies provided data on types of production systems, higher ESBL-*E. coli* proportions (23.8 – 93.8%) were reported in large-scale farms compared to the small and medium scale farms (0 - 35.3%). This can probably be explained by the tendency of large-scale intensive farms to utilize antibiotics at higher rates than farms operated on a small scale to prevent and treat infectious diseases and for growth promotion purposes (Manyi-Loh et al., 2018).

Two of the three studies conducted in backyard farms reported ESBL proportions ranging from 2.8 to 2.9%. Albeit low, the confirmation of ESBLs in this setting cannot be overlooked because backyard production systems have been associated with a greater risk of human exposure to resistant bacteria of poultry origin. This can be attributed to the frequent and close contact between poultry and humans who live in close quarters with these birds and consume poultry products directly from their keep (Graham et al., 2017; Alders et al., 2018). Regardless, this is not to trivialize the risk raised by large-scale commercial farms to consumers of products of poultry origin from these farms and the potential spread of AMR in the environment through animal waste derived from food production and use of poultry litter as fertilizer in crop production.

Only five studies (15.2%) reported the age of birds sampled with varying levels of ESBL s. However, the difference in reporting levels (counts and proportions), variety of samples used in the different studies, and few numbers of studies made it difficult to make meaningful comparisons to strongly associate age with ESBL levels. Most of the studies reported the sampled population as simply “poultry” or “chicken”. However, in the few that specified sampling broilers and layers, no major difference was noted in the average ESBL levels. This is in contrast with the findings from previous studies which reported higher levels of ESBLs in broiler (87% and 81%) compared with layer farms (42% and 65%) in Asia and Europe respectively (Blaak et al., 2015; Brower et al., 2017). Again, most of these ESBL proportions were estimated using non-probabilistic sampling and so it was not possible to extrapolate the true ESBL prevalence in these populations.

Most of the studies (26 studies, 78.8%) were published after 2015. However, this does not translate to increased detection of ESBLs from 2015 onwards because the study period often varied from publication year. On the other hand, there was a 50% reduction in the number of published studies in 2020 compared to the previous two years. The reduction can probably be explained by the preoccupation of the scientific community with building the evidence base during the COVID-19 pandemic, and a bias on the part of publishers who were more likely to publish novel findings of COVID-19 than other topics. Furthermore, the lockdown and travel restrictions resulting from the pandemic led to a closure of scientific workplaces and the interruption of field work, and consequently an extension of research time, re-starting some experiments, and putting some experiments and field activities on hold (Subramanya et al., 2020). There was also the issue of redirection of funding intended for other research areas to COVID-19 research (Chinnery et al., 2021).

### 4.1 Research gaps

Studies included in this review came from only nine out of 54 countries (16%) in Africa. Notably, no studies from Central and Southern Africa were found to be relevant to the scope of this review. While it can be argued that ESBLs are probably being assessed in other food-producing animals, or poultry-derived products, the dearth of publications related to ESBL-*E.coli* in poultry in African countries is evident. In addition, no surveillance reports assessing the trends of ESBL-producing *E. coli* from poultry in Africa were identified by this study. The World Health Organization (WHO) commissioned the Tricycle project, an integrated multisectoral surveillance project to monitor ESBL-producing *E. coli* across human, poultry, and environment sectors (WHO, 2021), that is currently being implemented in African countries such as Zimbabwe. This paucity of data hinders the assessment of the risk that ESBL-producing *E. coli* pose to both animals and humans in Africa.

In terms of sampling, the use of convenience and unspecified sampling strategies was observed in about 94% of studies. Unfortunately, this makes it difficult to make generalized inferences with the findings of these studies since the sampled population is not representative of the general poultry population. Consequently, it poses a challenge to policy makers because good quality data is required to inform effective and sustainable policies and interventions.

Finally, incomplete reporting of data in the studies especially those related to poultry populations (age, health status, specific poultry species), type of production systems, patterns, and extent of antibiotic use on farms, and ESBL levels prevents meaningful comparisons from being drawn and the identification of production systems that are at a higher risk of becoming exposed and colonized with ESBLs, therefore presenting a risk to consumers and dissemination of AMR into the environment.

### 4.2 Limitations of the study

The findings of this review should be interpreted bearing the following limitations. Due to the nature of this study, that was conducted as part of the requirements to fulfil a master’s degree in One Health, the study selection, screening, and data extraction processes were carried out by one reviewer with regular checks undertaken by the project supervisor. This increased the risk of bias while undertaking these steps as normally, study screening and data extraction would be conducted in parallel by two independent reviewers in systematic reviews. A protocol (ID: CRD42021259872) was developed for this systematic review *a priori* and submitted to PROSPERO (https://www.crd.york.ac.uk/prospero/) and the entire review process was carried out with strict adherence to the protocol. In addition, the entire screening process was documented, and all identified studies were uploaded in a shared Mendeley folder where the supervisor could access them for rechecks.

Only studies published in English were included in this review; this could have led to the exclusion of studies and reports from African countries where English is not one of the official languages (e.g., French- and Portuguese-speaking countries). The likelihood of excluding relevant data was also increased by the rejection of articles for which full texts could not be obtained. The findings of this review might have been influenced by these excluded studies.

This review excluded some studies which assessed poultry alongside other species, and others that assessed poultry feces alongside poultry-derived products when ESBL data was reported in aggregated form. This made it impossible to discern the proportion of the total ESBLs levels that could be attributed to the animal population of interest. In this review, studies that did not specify the type of poultry were included with the assumption that they met the inclusion criteria. However, there is no guarantee that ducks and geese were not sampled in these studies, and this may have added some bias to the review. Finally, the interpretation of findings was carried out with the data provided by studies with variable quality and levels of risk of bias. However, a quality and risk of bias assessment was conducted and only studies with moderate and low risk of bias were included in this review.

### 4.3 Implications of findings and recommendations

This review identified a major gap in the quantity and quality of evidence related to ESBL-*E. coli* of poultry origin in Africa. Due to the limited amount of data gathered by this review, it is recommended that additional research determining the prevalence of ESBL-*E. coli* and the diversity of ESBL genes circulating across sectors should be carried out following a One Health approach especially in the Southern and Central Africa regions. More attention should also be given to addressing risks of bias and controlling confounding in studies to build high-quality evidence base on which extrapolations can be made to inform development of effective policies and interventions.

The isolation of MDR ESBL-*E. coli* isolates in three regions in Africa emphasizes the urgent need to address the problem of AMR on the continent. From a socioeconomic perspective, the use of antibiotics for growth promotion is popular because it is done to obtain maximum yield from livestock production (Durso and Cook, 2014), hence protecting the livelihood of the farmer. However, the use of antibiotics at sub-therapeutic doses leads to selection of resistant bacteria in the intestinal flora of birds thereby contributing to AMR (Essack et al., 2017). In many African countries, antibiotics are often used to make up for loopholes in biosecurity and good animal husbandry practices (e.g., provision of good nutrition, vaccination) (Hedman et al., 2020). Therefore, the imposition of restrictions on ABU may have a negative impact on both animal health and welfare as well as the livelihood of farmers and local economies. Instead, farmers should be sensitized on the importance of implementing strict biosecurity measures and good animal husbandry practices, in order to prevent introduction of pathogens and therefore, reducing the burden of disease in their flocks and the need for antibiotics. This can be done by engaging farmers in experiential learning activities as seen in the FAO’s Farmer Field School (FFS) initiative (FAO, 2016).

This review identified ESBL genes in poultry that are also commonly isolated in humans, suggesting a potential risk of zoonotic transfer of ESBL-producing *E. coli*. However, the full extent of ESBL-producing *E. coli* and its zoonotic transmission are not yet fully understood due to the limited evidence available in Africa. Therefore, there is a need for more research employing a One Health approach, exploring AMR across sectors (humans, animals, and the environment) and adequate source attribution methods. These methods allow the identification of key hotspots where interventions are likely to be more effective in reducing the risk of AMR emergence and spread to humans and animals (Valentin et al., 2014). In addition, there is a need for increased awareness and engagement of key stakeholders in all key sectors to tackle ESBL-producing bacteria in Africa. In the development of national action plans, cooperation and collaboration between departments of veterinary services, and public health, the Government and all actors in the antibiotic supply chain is essential. Considerations should also be made with respect to resource allocation for the integrated surveillance of AMR across human, animal, and environmental sectors to generate data to support evidence-based policies and interventions for AMR.

## 5 Conclusions

The occurrence of ESBLs in poultry populations has been identified as a matter of public health importance worldwide given the zoonotic risk posed by these species to humans mainly through direct contact with birds and consumption of poultry-derived products. Synthesis of the available data revealed a frequent detection of ESBL-producing *E. coli* in poultry in Africa at varying levels across regions. The *bla*_CTX-M_ gene was identified as the most predominant gene family in this review. However, the full burden of ESBL-producing *E. coli* and its risks to humans and animals are not yet fully understood due to the limited evidence available in Africa. Further research addressing these gaps is therefore recommended.

## Author contributions

AA and AM: Conceptualization. AM: methodology. AM: validation. AA: formal analysis. AA: investigation. AA: writing—original draft preparation. AM: writing—review and editing. AM: visualization. AM: supervision. All authors contributed to the article and approved the submitted version.

## References

[B1] Adeleye E. O. Ayeni L. S. Ojeniyi S. (2010) Effect of poultry manure on soil physico-chemical properties, leaf nutrient contents and yield of yam (Dioscorea rotundata) on alfisol in southwestern Nigeria. Available at: http://www.Americanscience.orgeditor@americanscience.org.

[B2] Agabou A. Lezzar N. Ouchenane Z. Khemissi S. Satta D. Sotto A. . (2016). Clonal relationship between human and avian ciprofloxacin-resistant escherichia coli isolates in north-Eastern Algeria. Eur. J. Clin. Microbiol. Infect. Dis. 35 (2), 227–234. doi: 10.1007/s10096-015-2534-3 26634353

[B3] Alders R. G. Dumas S. E. Rukambile E. Magoke G. Maulaga W. Jong J. . (2018). Family poultry: multiple roles, systems, challenges, and options for sustainable contributions to household nutrition security through a planetary health lens. Maternal Child Nutr. 14 (Suppl 3). doi: 10.1111/MCN.12668 PMC6221142 30332533

[B4] Alonso C. A. Zarazaga M. ben Sallem R. Jouini A. ben Slama K. Torres C. (2017). Antibiotic resistance in escherichia coli in husbandry animals: the African perspective. Lett. Appl. Microbiol. 64 (5), 318–334). doi: 10.1111/lam.12724 28208218

[B5] Al-Orphaly M. Hadi H. A. Eltayeb F. K. Al-Hail H. Samuel B. G. Sultan A. A. . (2021). Epidemiology of multidrug-resistant pseudomonas aeruginosa in the middle East and north Africa region. MSphere 6 (3). doi: 10.1128/MSPHERE.00202-21 PMC8265635 34011686

[B6] Aworh M. K. Kwaga J. Okolocha E. Harden L. Hull D. Hendriksen R. S. . (2020). Extended-spectrum ß-lactamase-producing escherichia coli among humans, chickens and poultry environments in Abuja, Nigeria. One Health Outlook. 2 (1). doi: 10.1186/s42522-020-00014-7 PMC7993457 33829130

[B7] Ayandiran T. O. Falgenhauer L. Schmiede J. Chakraborty T. Ayeni F. A. (2018). High resistance to tetracycline and ciprofloxacin in bacteria isolated from poultry farms in ibadan, Nigeria. J. Infect. Develop. Countries. 12 (6), 462–470. doi: 10.3855/jidc.9862 31940298

[B8] Ayeni F. A. Olujobi O. F. Alabi S. (2015). A preliminary investigation of prevalence of extended spectrum beta lactamases among enterobacteriaceae isolated from poultry. Nigerian. J. Pharm. Res. 11 (1), 46–51. Available at: https://www.ajol.info/index.php/njpr/article/view/162992

[B9] Badi S. Cremonesi P. Abbassi M. S. Ibrahim C. Snoussi M. Bignoli G. . (2018). Antibiotic resistance phenotypes and virulence-associated genes in escherichia coli isolated from animals and animal food products in Tunisia. FEMS Microbiol. Lett. 365 (10). doi: 10.1093/femsle/fny088 29635468

[B10] Bajpai T. Pandey M. Varma M. Bhatambare G. S. (2017). Prevalence of TEM, SHV, and CTX-m beta-lactamase genes in the urinary isolates of a tertiary care hospital. Avicenna. J. Med. 7 (1), 12. doi: 10.4103/2231-0770.197508 28182026 PMC5255976

[B11] Belmahdi M. Bakour S. al Bayssari C. Touati A. Rolain J. M. (2016). Molecular characterisation of extended-spectrum β-lactamase- and plasmid AmpC-producing escherichia coli strains isolated from broilers in béjaïa, Algeria. J. Global Antimicrob. Resist. 6, 108–112. doi: 10.1016/j.jgar.2016.04.006 27530851

[B13] Benavides J. A. Shiva C. Virhuez M. Tello C. Appelgren A. Vendrell J. . (2018). Extended-spectrum beta-lactamase-producing escherichia coli in common vampire bats desmodus rotundus and livestock in Peru. Zoonoses. Public Health 65 (4), 454–458. doi: 10.1111/ZPH.12456 29575785 PMC6446800

[B12] ben Sallem R. ben Slama K. Sáenz Y. Rojo-Bezares B. Estepa V. Jouini A. . (2012). Prevalence and characterization of extended-spectrum beta-lactamase (ESBL)- and CMY-2-producing escherichia coli isolates from healthy food-producing animals in Tunisia. Foodborne. Pathog. Dis. 9 (12), 1137–1142. doi: 10.1089/fpd.2012.1267 23194332

[B14] Berendes D. Kirby A. Brown J. Wester A. L. (2020). Human faeces-associated extended-spectrum β-lactamase-producing escherichia coli discharge into sanitation systems in 2015 and 2030: a global and regional analysis. Lancet Planet. Health 4 (6), e246–e255. doi: 10.1016/S2542-5196(20)30099-1 32559441 PMC10906806

[B15] Blaak H. van Hoek A. H. A. M. Hamidjaja R. A. van der Plaats R. Q. J. Heer L. K. de Husman A. M. R. . (2015). Distribution, numbers, and diversity of ESBL-producing e. coli in the poultry farm environment. PloS One 10 (8). doi: 10.1371/JOURNAL.PONE.0135402 PMC4536194 26270644

[B16] Blanc V. Mesa R. Saco M. Lavilla S. Prats G. Miró E. . (2006). ESBL- and plasmidic class c β-lactamase-producing e. coli strains isolated from poultry, pig and rabbit farms. Vet. Microbiol. 118 (3–4), 299–304. doi: 10.1016/j.vetmic.2006.08.002 16973308

[B17] Braun S. D. Ahmed M. F. E. El-Adawy H. Hotzel H. Engelmann I. Wei D. . (2016). Surveillance of extended-spectrum beta-lactamase-producing escherichia coli in dairy cattle farms in the nile delta, Egypt. Front. Microbiol. 7 (JUL). doi: 10.3389/fmicb.2016.01020 PMC4931819 27458435

[B18] Briñas L. Moreno M. A. Zarazaga M. Porrero C. Sáenz Y. García M. . (2003). Detection of CMY-2, CTX-M-14, and SHV-12 β-lactamases in escherichia coli fecal-sample isolates from healthy chickens. Antimicrob. Agents Chemother. 47 (6), 2056. doi: 10.1128/AAC.47.6.2056-2058.2003 12760899 PMC155838

[B19] Brower C. H. Mandal S. Hayer S. Sran M. Zehra A. Patel S. J. . (2017). The prevalence of extended-spectrum beta-Lactamase-Producing multidrug-resistant escherichia coli in poultry chickens and variation according to farming practices in punjab, India. Environ. Health Perspect. 125 (7). doi: 10.1289/EHP292 PMC5744676 28749780

[B20] Büdel T. Kuenzli E. Campos-Madueno E. I. Mohammed A. H. Hassan N. K. Zinsstag J. . (2020). On the island of Zanzibar people in the community are frequently colonized with the same MDR enterobacterales found in poultry and retailed chicken meat. J. Antimicrob. Chemother. 75 (9), 2432–2441. doi: 10.1093/jac/dkaa198 32562537

[B21] Cantón R. Akóva M. Carmeli Y. Giske C. G. Glupczynski Y. Gniadkowski M. . (2012). Rapid evolution and spread of carbapenemases among enterobacteriaceae in Europe. Clin. Microbiol. Infect. 18 (5), 413–431). doi: 10.1111/j.1469-0691.2012.03821.x 22507109

[B22] Chabou S. Leulmi H. Davoust B. Aouadi A. Rolain J. M. (2018). Prevalence of extended-spectrum β-lactamase- and carbapenemase-encoding genes in poultry faeces from Algeria and marseille, France. J. Global Antimicrob. Resist. 13, 28–32. doi: 10.1016/j.jgar.2017.11.002 29138113

[B23] Chah K. F. Ugwu I. C. Okpala A. Adamu K. Y. Alonso C. A. Ceballos S. . (2018). Detection and molecular characterisation of extended-spectrum β-lactamase-producing enteric bacteria from pigs and chickens in nsukka, Nigeria. J. Global Antimicrob. Resist. 15, 36–40. doi: 10.1016/j.jgar.2018.06.002 29908916

[B24] Chinnery P. F. Pearce J. J. Kinsey A. M. Jenkinson J. M. Wells G. Watt F. M. (2021). How COVID-19 has changed medical research funding. Interface Focus 11 (6). doi: 10.1098/RSFS.2021.0025 PMC8504879 34956595

[B25] Depoorter P. Persoons D. Uyttendaele M. Butaye P. de Zutter L. Dierick K. . (2012). Assessment of human exposure to 3rd generation cephalosporin resistant e. coli (CREC) through consumption of broiler meat in Belgium. Int. J. Food Microbiol. 159 (1), 30–38. doi: 10.1016/j.ijfoodmicro.2012.07.026 22938836

[B26] Dierikx C. M. Goot J. A. Smith H. E. Kant A. Mevius D. J. (2013). Presence of ESBL/AmpC -producing escherichia coli in the broiler production pyramid: a descriptive study. PloS One 8 (11), e79005. doi: 10.1371/JOURNAL.PONE.0079005 24244401 PMC3820706

[B27] Dierikx C. van Duijkeren E. Schoormans A. van Essen-Zandbergen A. Veldman K. Kant A. . (2012). Occurrence and characteristics of extended-spectrum-β-lactamase- and AmpC-producing clinical isolates derived from companion animals and horses. J. Antimicrob. Chemother. 67 (6), 1368–1374. doi: 10.1093/JAC/DKS049 22382469

[B28] Donkor E. Newman M. Yeboah-Manu D. (2012). Epidemiological aspects of non-human antibiotic usage and resistance: implications for the control of antibiotic resistance in Ghana. Trop. Med. Int. Health.: TM. IH. 17 (4), 462–468. doi: 10.1111/J.1365-3156.2012.02955.X 22413809

[B29] Durso L. M. Cook K. L. (2014). Impacts of antibiotic use in agriculture: what are the benefits and risks? Curr. Opin. Microbiol. 19 (1), 37–44. doi: 10.1016/J.MIB.2014.05.019 24997398

[B30] EFSA (2011). Scientific opinion on the public health risks of bacterial strains producing extended-spectrum β-lactamases and/or AmpC β-lactamases in food and food-producing animals. EFSA. J. 9 (8). doi: 10.2903/J.EFSA.2011.2322

[B31] Elton L. Thomason M. J. Tembo J. Velavan T. P. Pallerla S. R. Arruda L. B. . (2020). Antimicrobial resistance preparedness in sub-Saharan African countries. Antimicrob. Resist. Infect. Control. 9 (1), 1–11. doi: 10.1186/S13756-020-00800-Y 32859252 PMC7456056

[B32] Essack S. Y. Desta A. T. Abotsi R. E. Agoba E. E. (2017). Antimicrobial resistance in the WHO African region: current status and roadmap for action. J. Public Health (Oxford. England). 39 (1), 8. doi: 10.1093/PUBMED/FDW015 PMC5939661 26944074

[B33] EUCAST (2021) EUCAST: clinical breakpoints and dosing of antibiotics. Available at: https://eucast.org/clinical_breakpoints/.

[B34] Ewers C. Bethe A. Semmler T. Guenther S. Wieler L. H. (2012). Extended-spectrum β-lactamase-producing and AmpC-producing escherichia coli from livestock and companion animals, and their putative impact on public health: a global perspective. Clin. Microbiol. Infect. 18 (7), 646–655. doi: 10.1111/j.1469-0691.2012.03850.x 22519858

[B35] Falgenhauer L. Imirzalioglu C. Oppong K. Akenten C. W. Hogan B. Krumkamp R. . (2019). Detection and characterization of ESBL-producing escherichia coli from humans and poultry in Ghana. Front. Microbiol. 10 (JAN). doi: 10.3389/fmicb.2018.03358 PMC6340976 30697208

[B36] FAO (2014). DECISION TOOLS FOR FAMILY POULTRY DEVELOPMENT enabling poor rural people to overcome poverty RURAL POULTRY CENTRE. Food and Agricultural Organization of the United Nations (FAO)

[B37] FAO (2016). FARMER FIELD SCHOOL GUIDANCE DOCUMENT planning for quality programmes plant production and protection division food and agriculture organization of the united nations viale delle terme di caracalla 00153 (Rome, Italy: Food and Agricultural Organization of the United Nations (FAO)). Available at: www.fao.org/ag/agp.

[B38] Fortini D. Fashae K. García-Fernández A. Villa L. Carattoli A. (2011). Plasmid-mediated quinolone resistance and β-lactamases in escherichia coli from healthy animals from Nigeria. J. Antimicrob. Chemother. 66 (6), 1269–1272. doi: 10.1093/jac/dkr085 21393162

[B39] Giriyapur R. S. Nandihal N. W. Krishna B. V. S. Patil A. B. Chandrasekhar M. R. (2011). Comparison of disc diffusion methods for the detection of extended-spectrum beta lactamase-producing enterobacteriaceae. J. Lab. Phys. 3 (1), 33. doi: 10.4103/0974-2727.78561 PMC3118054 21701661

[B40] Graham J. P. Eisenberg J. N. S. Trueba G. Zhang L. Johnson T. J. (2017). Small-scale food animal production and antimicrobial resistance: mountain, molehill, or something in-between? Environ. Health Perspect. 125 (10). doi: 10.1289/EHP2116 PMC5933306 29038091

[B41] Grami R. Mansour W. Dahmen S. Mehri W. Haenni M. Aouni M. . (2013). The blaCTX-M-1 IncI1/ST3 plasmid is dominant in chickens and pets in tunisia. J. Antimicrob. Chemother. 68 (12), 2950–2952). doi: 10.1093/jac/dkt258 23800900

[B42] Gundran R. S. Cardenio P. A. Villanueva M. A. Sison F. B. Benigno C. C. Kreausukon K. . (2019). Prevalence and distribution of bla CTX-m, bla SHV, bla TEM genes in extended- spectrum β- lactamase- producing e. coli isolates from broiler farms in the Philippines. BMC Vet. Res. 15 (1), 1–8. doi: 10.1186/S12917-019-1975-9 31277658 PMC6612079

[B43] Hassen B. Abbassi M. S. Ruiz-Ripa L. Mama O. M. Hassen A. Torres C. . (2020). High prevalence of mcr-1 encoding colistin resistance and first identification of blaCTX-M-55 in ESBL/CMY-2-producing escherichia coli isolated from chicken faeces and retail meat in Tunisia. Int. J. Food Microbiol. 318. doi: 10.1016/j.ijfoodmicro.2019.108478 31855787

[B44] Hedman H. D. Vasco K. A. Zhang L. (2020). A review of antimicrobial resistance in poultry farming within low-resource settings. Animals 10 (8), 1–39). doi: 10.3390/ani10081264 PMC7460429 32722312

[B45] Horcajada J. Montero M. Oliver A. Sorlí L. Luque S. Gómez-Zorrilla S. . (2019). Epidemiology and treatment of multidrug-resistant and extensively drug-resistant pseudomonas aeruginosa infections. Clin. Microbiol. Rev. 32 (4). doi: 10.1128/CMR.00031-19 PMC6730496 31462403

[B46] Huijbers P. M. C. Graat E. A. M. Haenen A. P. J. van Santen M. G. van Essen-Zandbergen A. Mevius D. J. . (2014). Extended-spectrum and AmpC β-lactamase-producing escherichia coli in broilers and people living and/or working on broiler farms: prevalence, risk factors and molecular characteristics. J. Antimicrob. Chemother. 69 (10), 2669–2675. doi: 10.1093/jac/dku178 24879667

[B47] Jena J. Sahoo R. K. Debata N. K. Subudhi E. (2017). Prevalence of TEM, SHV, and CTX-m genes of extended-spectrum β-lactamase-producing escherichia coli strains isolated from urinary tract infections in adults. 3. Biotech. 7 (4). doi: 10.1007/S13205-017-0879-2 PMC5511117 28710743

[B48] Jouini A. Vinué L. ben Slama K. Sáenz Y. Klibi N. Hammami S. . (2007). Characterization of CTX-m and SHV extended-spectrum β-lactamases and associated resistance genes in escherichia coli strains of food samples in Tunisia. J. Antimicrob. Chemother. 60 (5), 1137–1141. doi: 10.1093/jac/dkm316 17855726

[B49] Katakweba A. A. S. Muhairwa A. P. Lupindu A. M. Damborg P. Rosenkrantz J. T. Minga U. M. . (2018). First report on a randomized investigation of antimicrobial resistance in fecal indicator bacteria from livestock, poultry, and humans in Tanzania. Microb. Drug Resist. 24 (3), 260–268. doi: 10.1089/mdr.2016.0297 28759321

[B50] Kiiru J. Kariuki S. Goddeeris B. M. Butaye P. (2012). Analysis of β-lactamase phenotypes and carriage of selected β-lactamase genes among escherichia coli strains obtained from Kenyan patients during an 18-year period. BMC Microbiol. 12, 155. doi: 10.1186/1471-2180-12-155 22838634 PMC3464591

[B51] Kilani H. Abbassi M. S. Ferjani S. Mansouri R. Sghaier S. Salem R. . (2015). Occurrence of blaCTX-M-1, qnrB1 and virulence genes in avian ESBL-producing escherichia coli isolates from Tunisia. Front. Cell. Infect. Microbiol. 5 (MAY). doi: 10.3389/fcimb.2015.00038 PMC4419849 26000252

[B52] Kilani H. Ferjani S. Mansouri R. Boutiba-Benboubaker I. Abbassi M. S. (2020). Occurrence of plasmid-mediated quinolone resistance determinants among escherichia coli strains isolated from animals in Tunisia: specific pathovars acquired qnr genes. J. Global Antimicrob. Resist. 20, 50–55. doi: 10.1016/j.jgar.2019.07.023 31365855

[B53] Kimera Z. I. Mgaya F. X. Misinzo G. Mshana S. E. Moremi N. Matee M. I. N. (2021). Multidrug-resistant, including extended-spectrum beta lactamase-producing and quinolone-resistant, escherichia coli isolated from poultry and domestic pigs in dar es salaam, Tanzania. Antibiotics 10 (4), 406. doi: 10.3390/antibiotics10040406 33918543 PMC8069735

[B54] Klein E. Y. Boeckel T. P. Martinez E. M. Pant S. Gandra S. Levin S. A. . (2018). Global increase and geographic convergence in antibiotic consumption between 2000 and 2015. Proc. Natl. Acad. Sci. United. States America 115 (15), E3463. doi: 10.1073/PNAS.1717295115 PMC5899442 29581252

[B55] Kwoji I. D. Musa J. A. Daniel N. Mohzo D. L. Bitrus A. A. Ojo A. A. . (2019). Extended-spectrum beta-lactamase-producing escherichia coli in chickens from small-scale (backyard) poultry farms in maiduguri, Nigeria. Int. J. One Health 5, 26–30. doi: 10.14202/IJOH.2019.26-30

[B56] Langata L. M. Maingi J. M. Musonye H. A. Kiiru J. Nyamache A. K. (2019). Antimicrobial resistance genes in salmonella and escherichia coli isolates from chicken droppings in Nairobi, Kenya. BMC Res. Notes 12 (1). doi: 10.1186/s13104-019-4068-8 PMC6332563 30642404

[B57] Laube H. Friese A. von Salviati C. Guerra B. Käsbohrer A. Kreienbrock L. . (2013). Longitudinal monitoring of extended-spectrum-beta-lactamase/ampC-producing escherichia coli at german broiler chicken fattening farms. Appl. Environ. Microbiol. 79 (16), 4815–4820. doi: 10.1128/AEM.00856-13 23747697 PMC3754693

[B58] Li Y. Chen L. Wu X. Huo S. (2015). Molecular characterization of multidrug-resistant avian pathogenic escherichia coli isolated from septicemic broilers. Poult. Sci. 94 (4), 601–611. doi: 10.3382/PS/PEV008 25667425

[B59] Maamar E. Hammami S. Alonso C. A. Dakhli N. Abbassi M. S. Ferjani S. . (2016). High prevalence of extended-spectrum and plasmidic AmpC beta-lactamase-producing escherichia coli from poultry in Tunisia. Int. J. Food Microbiol. 231, 69–75. doi: 10.1016/j.ijfoodmicro.2016.05.001 27220012

[B60] Magiorakos A. Srinivasan A. Carey R. Carmeli Y. Falagas M. Giske C. . (2012). Multidrug-resistant, extensively drug-resistant and pandrug-resistant bacteria: an international expert proposal for interim standard definitions for acquired resistance. Clin. Microbiol. Infect. 18 (3), 268–281. doi: 10.1111/J.1469-0691.2011.03570.X 21793988

[B61] Manyi-Loh C. Mamphweli S. Meyer E. Okoh A. (2018). Antibiotic use in agriculture and its consequential resistance in environmental sources: potential public health implications. Mol. : A. J. Synthetic. Chem. Natural Product. Chem. 23 (4). doi: 10.3390/MOLECULES23040795 PMC6017557 29601469

[B62] Maron D. F. Smith T. J. Nachman K. E. (2013). Restrictions on antimicrobial use in food animal production: an international regulatory and economic survey. Globalization. Health 9 (1), 48. doi: 10.1186/1744-8603-9-48 24131666 PMC3853314

[B63] Meguenni N. Chanteloup N. Tourtereau A. Ahmed C. A. Bounar-Kechih S. Schouler C. (2019). Virulence and antibiotic resistance profile of avian escherichia coli strains isolated from colibacillosis lesions in central of Algeria. Vet. World 12 (11), 1840–1848. doi: 10.14202/vetworld.2019.1840-1848 32009764 PMC6925048

[B64] Messaili C. Messai Y. Bakour R. (2019). Virulence gene profiles, antimicrobial resistance and phylogenetic groups of fecal escherichia coli strains isolated from broiler chickens in algeria. Vet. Italiana. 55 (1), 35–46. doi: 10.12834/VetIt.799.3865.2 30951180

[B65] Mgaya F. X. Matee M. I. Muhairwa A. P. Hoza A. S. (2021). Occurrence of multidrug ResistantEscherichia coliin raw meatand cloaca swabs in poultry processed in slaughter slabs inDar es salaam, Tanzania. Antibiotics 10 (4), 343. doi: 10.3390/antibiotics10040343 33804812 PMC8063811

[B66] Mnif B. Ktari S. Rhimi F. M. Hammami A. (2012). Extensive dissemination of CTX-M-1- and CMY-2-producing escherichia coli in poultry farms in Tunisia. Lett. Appl. Microbiol. 55 (6), 407–413. doi: 10.1111/j.1472-765X.2012.03309.x 22966763

[B67] Moawad A. A. Hotzel H. Neubauer H. Ehricht R. Monecke S. Tomaso H. . (2018). Antimicrobial resistance in enterobacteriaceae from healthy broilers in Egypt: emergence of colistin-resistant and extended-spectrum β-lactamase-producing escherichia coli 06 biological sciences 0604 genetics 11 medical and health sciences 1108 medical mi. Gut. Pathog. 10 (1). doi: 10.1186/s13099-018-0266-5 PMC6148799 30250514

[B69] Mshana S. E. Falgenhauer L. Mirambo M. M. Mushi M. F. Moremi N. Julius R. . (2016). Predictors of blaCTX-M-15 in varieties of escherichia coli genotypes from humans in community settings in mwanza, Tanzania. BMC Infect. Dis. 16 (1). doi: 10.1186/S12879-016-1527-X PMC4850702 27129719

[B70] Mughini-Gras L. Dorado-García A. van Duijkeren E. van den Bunt G. Dierikx C. M. Bonten M. J. M. . (2019). Attributable sources of community-acquired carriage of escherichia coli containing β-lactam antibiotic resistance genes: a population-based modelling study. Lancet Planet. Health 3 (8), e357–e369. doi: 10.1016/S2542-5196(19)30130-5 31439317

[B71] Musa B. M. Imam H. Lendel A. Abdulkadir I. Gumi H. S. Aliyu M. H. . (2020). The burden of extended-spectrum β-lactamase-producing enterobacteriaceae in Nigeria: a systematic review and meta-analysis. Trans. R. Soc. Trop. Med. Hygiene. 114 (4), 241–248. doi: 10.1093/TRSTMH/TRZ125 31925440

[B72] Ojo O. E. Schwarz S. Michael G. B. (2016). Detection and characterization of extended-spectrum β-lactamase-producing escherichia coli from chicken production chains in Nigeria. Vet. Microbiol. 194, 62–68. doi: 10.1016/j.vetmic.2016.04.022 27157499

[B73] Okpara E. O. Ojo O. E. Awoyomi O. J. Dipeolu M. A. Oyekunle M. A. Schwarz S. (2018). Antimicrobial usage and presence of extended-spectrum β-lactamase-producing enterobacteriaceae in animal-rearing households of selected rural and peri-urban communities. Vet. Microbiol. 218, 31–39. doi: 10.1016/j.vetmic.2018.03.013 29685218

[B74] Okubo T. Yossapol M. Maruyama F. Wampande E. M. Kakooza S. Ohya K. . (2019). Phenotypic and genotypic analyses of antimicrobial resistant bacteria in livestock in Uganda. Transboundary. Emerging. Dis. 66 (1), 317–326. doi: 10.1111/tbed.13024 30260584

[B75] Olowe O. A. Adewumi O. Odewale G. Ojurongbe O. Adefioye O. J. (2015). Phenotypic and molecular characterisation of extended-spectrum beta-lactamase producing escherichia coli obtained from animal fecal samples in ado ekiti, Nigeria. J. Environ. Public Health 2015. doi: 10.1155/2015/497980 PMC4568380 26417371

[B76] Olsen R. H. Bisgaard M. Löhren U. Robineau B. Christensen H. (2014). Extended-spectrum β-lactamase-producing escherichia coli isolated from poultry: a review of current problems, illustrated with some laboratory findings. Avian Pathol. 43 (3), 199–208). doi: 10.1080/03079457.2014.907866 24666286

[B77] Overdevest I. Willemsen I. Rijnsburger M. Eustace A. Xu L. Hawkey P. . (2011). Extended-spectrum β-lactamase genes of escherichia coli in chicken meat and humans, the Netherlands. Emerging. Infect. Dis. 17 (7), 1216. doi: 10.3201/EID1707.110209 PMC3381403 21762575

[B78] Page M. J. McKenzie J. E. Bossuyt P. M. Boutron I. Hoffmann T. C. Mulrow C. D. . (2021) The PRISMA 2020 statement: an updated guideline for reporting systematic reviews. BMJ 372. doi: 10.1136/BMJ.N71 PMC8005924 33782057

[B79] Pakzad I. Ghafourian S. Taherikalani M. sadeghifard N. Abtahi H. Rahbar M. . (2011). Qnr prevalence in extended spectrum beta-lactamases (ESBLs) and none-ESBLs producing escherichia coli isolated from urinary tract infections in central of Iran. Iranian. J. Basic. Med. Sci. 14 (5), 458. Available at: https://www.ncbi.nlm.nih.gov/pmc/articles/PMC3586849/ PMC3586849 23493061

[B80] Paterson D. L. Bonomo R. A. (2005). Extended-spectrum β-lactamases: a clinical update. Clin. Microbiol. Rev. 18 (4), 657. doi: 10.1128/CMR.18.4.657-686.2005 16223952 PMC1265908

[B81] Platell J. Johnson J. Cobbold R. Trott D. (2011). Multidrug-resistant extraintestinal pathogenic escherichia coli of sequence type ST131 in animals and foods. Vet. Microbiol. 153 (1–2), 99–108. doi: 10.1016/J.VETMIC.2011.05.007 21658865

[B82] Poirel L. Naas T. Nordmann P. (2010). Diversity, epidemiology, and genetics of class d β-lactamases. Antimicrob. Agents Chemother. 54 (1), 24. doi: 10.1128/AAC.01512-08 19721065 PMC2798486

[B83] Potron A. Poirel L. Nordmann P. (2015). Emerging broad-spectrum resistance in pseudomonas aeruginosa and acinetobacter baumannii: mechanisms and epidemiology. Int. J. Antimicrob. Agents 45 (6), 568–585. doi: 10.1016/J.IJANTIMICAG.2015.03.001 25857949

[B1000] Ramadan H. H. Jackson C. R. Taha S. A. Moawad A. A. Barrett J. B. Woodley T. A. (2018). Contribution of healthy chickens to antimicrobial-resistant escherichia coli associated with human extraintestinal infections in egypt. Vector Borne Zoonotic Dis. 18 (8), 408–416. doi: 10.1089/vbz.2017.2237 29927724

[B84] Ramos S. Silva V. Dapkevicius M. de L. E. Caniça M. Tejedor-Junco M. T. . (2020). Escherichia coli as commensal and pathogenic bacteria among food-producing animals: health implications of extended spectrum β-lactamase (ESBL) production. Animals.: Open Access J. MDPI. 10 (12), 1–15. doi: 10.3390/ANI10122239 PMC7761174 33260303

[B85] Rawat D. Nair D. (2010). Extended-spectrum β-lactamases in gram negative bacteria. J. Global Infect. Dis. 2 (3), 263. doi: 10.4103/0974-777X.68531 PMC2946684 20927289

[B86] Saidani M. Messadi L. Chaouechi A. Tabib I. Saras E. Soudani A. . (2019). High genetic diversity of enterobacteriaceae clones and plasmids disseminating resistance to extended-spectrum cephalosporins and colistin in healthy chicken in Tunisia. Microb. Drug Resist. 25 (10), 1507–1513. doi: 10.1089/mdr.2019.0138 31329501

[B87] Saino H. Sugiyabu T. Ueno G. Yamamoto M. Ishii Y. Miyano M. (2015). Crystal structure of OXA-58 with the substrate-binding cleft in a closed state: insights into the mobility and stability of the OXA-58 structure. PloS One 10 (12), e0145869. doi: 10.1371/JOURNAL.PONE.0145869 26701320 PMC4689445

[B1001] Sargeant J. M. Amezcua M. D. Waddell L. A. (2005). A guide to conducting systematic reviews in agri-food public health. Medicine.

[B1002] Sghaier S. Abbassi M. S. Pascual A. Serrano L. Díaz-De-Alba P. Said M. B. . (2019). Extended-spectrum β-lactamase-producing enterobacteriaceae from animal origin and wastewater in tunisia: first detection of O25b-B23-CTX-M-27-ST131 escherichia coli and CTX-M-15/OXA-204-producing citrobacter freundii from wastewater. J. Glob Antimicrob. Resist. 17, 189–194. doi: 10.1016/j.jgar.2019.01.002 30639890

[B88] Sharma M. Pathak S. Srivastava P. (2013). Prevalence and antibiogram of extended spectrum β-lactamase (ESBL) producing gram negative bacilli and further molecular characterization of ESBL producing escherichia coli and klebsiella spp. J. Clin. Diagn. Res. : JCDR 7 (10), 2173. doi: 10.7860/JCDR/2013/6460.3462 24298468 PMC3843424

[B89] Soliman A. M. Ramadan H. Sadek M. Nariya H. Shimamoto T. Hiott L. M. . (2020). Draft genome sequence of a blaNDM-1- and blaOXA-244-carrying multidrug-resistant escherichia coli d-ST69 clinical isolate from Egypt Vol. 22 (Elsevier Ltd), 832–834). doi: 10.1016/j.jgar.2020.07.015 32738341

[B90] Storberg V. (2014). ESBL-producing enterobacteriaceae in Africa – a non-systematic literature review of research published 2008–2012. Infect. Ecol. Epidemiol. 4 (1). doi: 10.3402/IEE.V4.20342 PMC3955770 24765249

[B91] Subramanya S. H. Lama B. Acharya K. P. (2020). Impact of COVID-19 pandemic on the scientific community. Qatar. Med. J. 2020 (1). doi: 10.5339/QMJ.2020.21 PMC7372729 32733784

[B92] Tängdén T. Cars O. Melhus A. Löwdin E. (2010). Foreign travel is a major risk factor for colonization with escherichia coli producing CTX-m-type extended-spectrum beta-lactamases: a prospective study with Swedish volunteers. Antimicrob. Agents Chemother. 54 (9), 3564–3568. doi: 10.1128/AAC.00220-10 20547788 PMC2934993

[B93] Tansawai U. Walsh T. R. Niumsup P. R. (2019). Extended spectrum ß-lactamase-producing escherichia coli among backyard poultry farms, farmers, and environments in Thailand. Poult. Sci. 98 (6), 2622–2631. doi: 10.3382/PS/PEZ009 30690545

[B94] Tufa T. B. Fuchs A. Tufa T. B. Stötter L. Kaasch A. J. Feldt T. . (2020). High rate of extended-spectrum beta-lactamase-producing gram-negative infections and associated mortality in Ethiopia: a systematic review and meta-analysis. Antimicrob. Resist. Infect. Control. 9 (1), 1–10. doi: 10.1186/S13756-020-00782-X 32771059 PMC7414654

[B95] Umair M. Mohsin M. Ali Q. Qamar M. Raza S. Ali A. . (2019). Prevalence and genetic relatedness of extended spectrum-β-Lactamase-Producing escherichia coli among humans, cattle, and poultry in Pakistan. Microb. Drug Resist. (Larchmont. N.Y.) 25 (9), 1374–1381. doi: 10.1089/MDR.2018.0450 31268408

[B96] UN (2011) UNSD — methodology. Available at: https://unstats.un.org/unsd/methodology/m49/.

[B97] Ur Rahman S. Ali T. Ali I. Khan N. A. Han B. Gao J. (2018). The growing genetic and functional diversity of extended spectrum beta-lactamases. BioMed. Res. Int. 2018. doi: 10.1155/2018/9519718 PMC5892270 29780833

[B99] Valentin L. Sharp H. Hille K. Seibt U. Fischer J. Pfeifer Y. . (2014). Subgrouping of ESBL-producing escherichia coli from animal and human sources: an approach to quantify the distribution of ESBL types between different reservoirs. Int. J. Med. Microbiol. 304 (7), 805–816. doi: 10.1016/J.IJMM.2014.07.015 25213631

[B100] Vounba P. Arsenault J. Bada-Alambédji R. Fairbrother J. M. (2019). Prevalence of antimicrobial resistance and potential pathogenicity, and possible spread of third generation cephalosporin resistance, in escherichia coli isolated from healthy chicken farms in the region of Dakar, Senegal. PloS One 14 (3). doi: 10.1371/journal.pone.0214304 PMC6435184 30913237

[B101] WHO (2021) WHO integrated global surveillance on ESBL-producing e. coli using a “One health” approach. Available at: https://www.who.int/publications/i/item/who-integrated-global-surveillance-on-esbl-producing-e.-coli-using-a-one-health-approach.

[B102] Zhao W. Hu Z. (2013). Epidemiology and genetics of CTX-m extended-spectrum β-lactamases in gram-negative bacteria. Crit. Rev. Microbiol. 39 (1), 79–101. doi: 10.3109/1040841X.2012.691460 22697133 PMC4086240

