# Supplementary material for: Extended-spectrum beta-lactamases in poultry in Africa: a systematic review
Source: Front Antibiot. 2023 May 8;2:1140750. doi: 10.3389/frabi.2023.1140750 (PMC11732038; doi:10.3389/frabi.2023.1140750)
Supplement: Supplementary file 1 [file Table_1.docx]

Supplementary Material

Extended-Spectrum Beta-Lactamases (ESBLs) in Poultry in Africa: A Systematic Review

Akeemat Ayinla*, Ana L. P. Mateus

*** Correspondence:** Akeemat Ayinla: hakimattayinla@gmail.com

# Supplementary Tables

**Supplementary Table S1: List of free text terms and MeSH headings utilized for the review**

| **MeSH terms** | | | |
| --- | --- | --- | --- |
| **Population** | **Exposure** | **Outcome** | **Situation** |
| Fowls, Domestic |  | Antibiotic Resistance  Antibiotic Resistance, Microbial  Antimicrobial Drug Resistance | No relevant MeSH terms |
| **Free text terms** |  |  |  |
| Poultry | ESBL | TEM OR SHV OR CTX-M OR OXA | Africa |
| Chicken | Extended spectrum beta lactamases | Cefotaxime | African continent |
| Broiler | Extended spectrum β–lactamases | Ceftazidime | Algeria |
| Turkey | Enterobacteria |  | Angola |
| Domestic birds | Escherichia coli |  | Benin |
|  | E. coli |  | Botswana |
|  |  |  | Burkina Faso |
|  |  |  | Burundi |
|  |  |  | Cabo Verde |
|  |  |  | Cameroon |
|  |  |  | Central African Republic (CAR) |
|  |  |  | Chad |
|  |  |  | Comoros |
|  |  |  | Democratic Republic of the Congo OR DRC |
|  |  |  | Republic of the Congo |
|  |  |  | Cote d'Ivoire OR Ivory Coast |
|  |  |  | Djibouti |
|  |  |  | Egypt |
|  |  |  | Equatorial Guinea |
|  |  |  | Eritrea |
|  |  |  | Eswatini OR Swaziland |
|  |  |  | Ethiopia |
|  |  |  | Gabon |
|  |  |  | Gambia |
|  |  |  | Ghana |
|  |  |  | Guinea |
|  |  |  | Guinea-Bissau |
|  |  |  | Kenya |
|  |  |  | Lesotho |
|  |  |  | Liberia |
|  |  |  | Libya |
|  |  |  | Madagascar |
|  |  |  | Malawi |
|  |  |  | Mali |
|  |  |  | Mauritania |
|  |  |  | Mauritius |
|  |  |  | Morocco |
|  |  |  | Mozambique |
|  |  |  | Namibia |
|  |  |  | Niger |
|  |  |  | Nigeria |
|  |  |  | Rwanda |
|  |  |  | Sao Tome and Principe |
|  |  |  | Senegal |
|  |  |  | Seychelles |
|  |  |  | Sierra Leone |
|  |  |  | Somalia |
|  |  |  | South Africa |
|  |  |  | South Sudan |
|  |  |  | Sudan |
|  |  |  | Tanzania |
|  |  |  | Togo |
|  |  |  | Tunisia |
|  |  |  | Uganda |
|  |  |  | Zambia |
|  |  |  | Zimbabwe |

**Supplementary Table S2: Quality Assessment Tool**

| S/N | DOMAINS | Response | Score | Instruction |
| --- | --- | --- | --- | --- |
| **Study objectives** | | | | |
| 1. | Do the objectives address the systematic review question? | Yes  No | 2  0 | Yes: Objectives clearly address the question  No: Objectives do not address the question |
| 2. | Was the sample size justified? | Yes  Partial  No | 2  1  0 | Yes: Use of sample-size formulas, based on  desired power or precision and estimate of  expected variability to detect differences.  Partially: Informal guesses of a sample size.  No: No details in the text. |
| **Outcome Assessment** | | | | |
| 3. | Were both screening and confirmatory phenotypic resistance detection methods described adequately? | Yes  Partial  No | 2  1  0 | Yes: Both screening and confirmatory tests adequately described  Partial: Only one of screening or confirmatory tests described  No: No phenotypic test described |
| 4. | Was the MIC^1^ reading adequately done following guidelines from accreditation systems? | Yes  Partial  No | 2  1  0 | Yes: MIC readings were done in line with CLSI^2^ (S ≤1 mg/L / R ≥4 mg/L for cefotaxime; S ≤4 mg/L / R ≥16 mg/L for ceftazidime)  or EUCAST^3^ (S ≤1 mg/L / R >2 mg/L for cefotaxime; S ≤1 mg/L / R >4 mg/L for ceftazidime)  guidelines  Partial: MIC readings were done in line with the guidelines of other accreditation bodies.  No: MIC reading not provided. |
| 5. | Were Genotypic resistance detection methods described adequately? | Yes  No | 2  0 | Yes: PCR^4^ and sequencing or DNA arrays described.  No: No genotypic test described |
| **Data Analysis** | | | | |
| 6. | Was the statistical analysis appropriate? | Yes  No | 2  0  2 | Yes: Analysis fits design, appropriate analysis  of clustered data when required.  No: No appropriate analysis described. |
| 7. | Were confounders appropriately considered? | Yes  Partial  No | 2  1  0  2 | Yes: Confounders were considered by exclusion, matching or  analytical control.  Partial: Some confounders controlled but  not all of those identify as important.  No |
| **Results** | | | | |
| 8. | Are the findings adequately reported in alignment with research objectives? | Yes  Partial  No | 2  1  0 | Yes: Findings are adequately reported.  Partial: A partial/biased report of the findings have been provided.  No: Findings are not adequately reported. |
| **Conclusions** | | | | |
| 9. | Were conclusions supported by the results? | Yes  No | 2  0 | Yes  No |

1: Minimum inhibitory concentration

2: Clinical and Laboratory Standards Institute

3: European Committee on Antimicrobial Susceptibility Testing

4: Polymerase chain reaction
